# Electro-optic cavities for in-situ measurement of cavity fields

**DOI:** 10.1038/s41377-024-01685-x

**Published:** 2025-02-06

**Authors:** Michael S. Spencer, Joanna M. Urban, Maximilian Frenzel, Niclas S. Mueller, Olga Minakova, Martin Wolf, Alexander Paarmann, Sebastian F. Maehrlein

**Affiliations:** 1 Department of Physical Chemistry, Fritz Haber Institute of the Max Planck Society, 14195 Berlin, Germany; 2 Institute of Radiation Physics, Helmholtz Zentrum Dresden Rossendorf, 01328 Dresden, Germany; 3 Institute of Applied Physics, Technische Universität Dresden, 01062 Dresden, Germany

**Keywords:** Imaging and sensing, Nonlinear optics, Terahertz optics, Polaritons

## Abstract

Cavity electrodynamics offers a unique avenue for tailoring ground-state material properties, excited-state engineering, and versatile control of quantum matter. Merging these concepts with high-field physics in the terahertz (THz) spectral range opens the door to explore low-energy, field-driven cavity electrodynamics, emerging from fundamental resonances or order parameters. Despite this demand, leveraging the full potential of field-driven material control in cavities is hindered by the lack of direct access to the intra-cavity fields. Here, we demonstrate a new concept of active cavities, consisting of electro-optic Fabry-Pérot resonators, which measure their intra-cavity electric fields on sub-cycle timescales. We thereby demonstrate quantitative retrieval of the cavity modes in amplitude and phase, over a broad THz frequency range. To enable simultaneous intra-cavity sampling alongside excited-state material control, we design a tunable multi-layer cavity, enabling deterministic design of hybrid cavities for polaritonic systems. Our theoretical models reveal the origin of the avoided crossings embedded in the intricate mode dispersion, and will enable fully-switchable polaritonic effects within arbitrary materials hosted by the hybrid cavity. Electro-optic cavities (EOCs) will therefore serve as integrated probes of light-matter interactions across all coupling regimes, laying the foundation for field-resolved intra-cavity quantum electrodynamics.

## Abstract (web summary)

We design, implement, and model electro-optic cavities (EOCs) to unlock time-domain measurements of intra-cavity fields. Furthermore, we decode avoided crossings in continuously tunable EOCs, enabling switchable light-matter coupling in the future.

## Graphical abstract

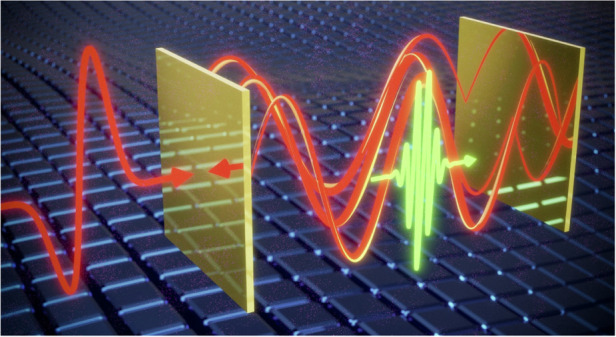

## Introduction

In recent decades, electromagnetic cavities have been a focus of intense research interest, providing experimental verification of cavity quantum electrodynamics principles, such as Bose-Einstein condensation in condensed matter^1^ and atomic systems^2^, as well as control over quantum-entangled cavity states^3^. Cavity electrodynamics has even been extended to on-chip photonic cavities^4,5^, along with demonstrations of complex cavity-coupled transport^6^ and chemistry^7^. Although these developments lead to significant impact across numerous scientific disciplines, they primarily focus on the visible (VIS), infrared (IR), and microwave spectral regions, corresponding to cavity eigenenergies outside the range of the majority of condensed-matter excitations. These fundamental, low-energy excitations are driven instead by picosecond (ps) electric field variations, and are therefore native to the ps^-1^ = 1 THz spectral region. Recent advances of carrier-envelope-phase-stable, high-field THz sources—spanning few to tens of THz, with peak field strengths on the order of 1–100 MV/cm, respectively^8–10^—open the door to high-field studies in the THz spectral region, thus setting the stage for fundamental investigations of cavity quantum electrodynamics.

The objectives of contemporary THz-cavity research are two-fold: Firstly, achieving cavity-induced renormalization of equilibrium material properties^11–14^, in the absence of external field driving, and secondly leveraging the resonant interactions between driven cavity modes and an active material, towards ultrafast control and dynamic design of coherently-driven, non-equilibrium states^15^. Cavity-induced renormalization of ground-state material properties has been explored in numerous theoretical works^11,12^, and has recently seen experimental verification in a strongly altered insulator-to-metal transition in 1T-TaS_2_^14^. Theoretical proposals include the transition from a quantum paraelectric to ferroelectric ground state in SrTiO_3_^12^, an anti-ferromagnetic to ferromagnetic transition in α-RuCl_3_^11^, and cavity-enhanced electron-electron attractions in pursuit of enhanced superconductivity^13,16,17^. The second class of proposals aim to leverage strongly-coupled light-matter states, termed polaritons, which are formed when the light-matter energy exchange rate exceeds the total system’s decoherence rate. Broadband, coherent excitation spanning these new resonances leads to time-domain beating at the Rabi frequency, as observed by coupling cavity modes to either phononic^14,18–22^ or magnonic^23–25^ resonances. These new states are proposed as promising handles for controlling high-field interactions, such as nonlinear phononics^15^, or enhanced phonon-driven superconductivity^26^. Further notable polaritonic effects can be accessed using near-field approaches, for targeting materials with intrinsic polaritons^27,28^. In this work, we focus on bulk cavities, which are readily accessible using far-field radiation, thereby conserving not only momentum, but also complex polarization states of tailored light^29^.

Although numerous applications of THz cavity physics have been proposed, the question remains, how can we best exploit the powerful tools of sub-cycle THz physics in the context of cavity electrodynamics? Most importantly, THz time-domain spectroscopy allows for the direct retrieval of amplitude- and phase-resolved electric fields^30^. This technique is particularly well suited to identify polaritonic effects, where strong light-matter coupling is directly observed, evidenced by Rabi oscillations between the polariton eigenstates^18,19,23–25^. Nevertheless, in all THz cavity measurements conducted to date, light-matter coupling has been assessed in an indirect way, by studying light that’s emitted from the cavity, i.e. after the fundamental process of light-matter coupling has occurred. THz-based techniques have thus been used as an extension of conventional VIS and near-IR techniques, unlocking amplitude-^19^ and phase-resolved^31^ cavity mode information. However, these studies suffer from several limitations: the transmitted electric field is significantly weaker compared to the intra-cavity fields, and is distorted after traveling through dispersive cavity mirrors. Furthermore, the mirror substrates introduce reflections^19,32^, whose destructive interference can be detrimental to coherent driving. These limitations can be overcome if the electric field is instead measured inside the cavity itself. It is therefore of fundamental scientific interest to enable in-situ measurement of cavity electric fields to unlock the full potential of THz cavity electrodynamics, enabling sub-cycle, and local measurements of exotic light-matter coupling states, directly where and when they are emerging within the cavity.

In this work, we demonstrate the amplitude- and phase-resolved measurement of intra-cavity fields. For this purpose, we develop a versatile platform of electro-optic cavities (EOCs), by integrating an electro-optic active medium within a Fabry-Pérot cavity. We develop a simple cavity correction function, accounting for both linear and nonlinear dispersive effects, as well as further local and non-local sampling effects, allowing us to extract the quantitative cavity electric fields. We establish EOCs across various cavity lengths and quality factors, and furthermore prototype a continuously-tunable hybrid EOC—a cavity consisting of a pair of electro-optic crystals separated by a tunable air gap. The latter structure surprisingly exhibits avoided crossings of its cavity modes, a typical signature of strongly-coupled oscillators, observed here without the inclusion of an additional active material. To understand this behavior, we develop a cavity field model, and a complementary coupled-oscillator model, which together explain these surprising observations. This detailed understanding directly informs deterministic design of hybrid EOCs, offering in-situ field sampling and tailored material interactions across all light-matter coupling regimes, while operating on sub-cycle time scales.

## Results

### Measuring intra-cavity fields

**Fig. 1:**
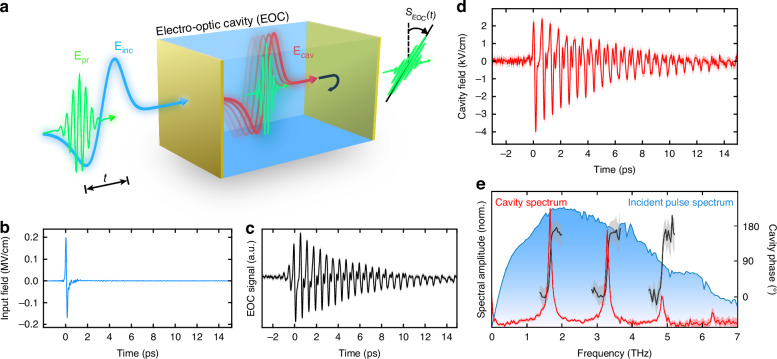
Electro-optic measurement of intra-cavity fields. **a** The cavity THz electric field (red) induces a local, transient birefringence as it travels inside an α-quartz electro-optic cavity (EOC), which is read out from the transmitted polarization state of an ultrashort co-propagating probing pulse (green), as a function of the relative time delay $t$ with respect to the incident single-cycle THz pulse (blue). **b** Incident ultra-broadband single-cycle THz field, measured by free-space EO sampling in z-cut α-quartz. **c** Intra-cavity EO signal generated from the input THz pulse in panel (**b**), for a quartz cavity length of 44 µm, and a nominal deposited gold mirror thickness of 14 nm. The standard error is represented here, and in panels (**d**, **e**), as a lighter area. **d** Quantitative, intra-cavity electric field derived from the inverse Fourier transform of the complex cavity spectrum in (**e**). **e** Normalized cavity spectrum derived from the measured EOC signal in (**c**) after applying the cavity correction function. Amplitude (red) and frequency-resolved phase (gray) are shown in comparison to the normalized spectrum of the incident broadband pulse in panel (**b**)

We demonstrate in Fig. 1, to our knowledge, the first direct measurement of the phase-resolved fields inside a Fabry-Pérot cavity. Here, we use the simplest design of an EOC, where the inversion-symmetry broken crystal fully fills the cavity, and its end facets are coated with thin gold films, functioning as the cavity end mirrors. This compact design allows for accurate and sensitive measurement of cavity fields, as depicted schematically in Fig. 1a, while simultaneously eliminating complications arising from end-mirror substrates^19,33^. The cavity field is driven by intense, single-cycle THz pump pulses (see Fig. 1b), and is probed with synchronized, 20 fs VIS pulses (see Methods M1). The probing of intra-cavity fields is achieved via the linear electro-optic (EO) effect (Pockels effect), where the THz field induces an effective transient birefringence according to its local amplitude, which is then experienced by the probe pulse^34^. The EO effect is mediated by the 2^nd^-order nonlinear susceptibility of the inversion-symmetry-broken electro-optic crystal. The resulting transient birefringence is extracted from the transmitted probe pulse polarization via a balanced-detection scheme (Fig. S9), as a function of pump-probe delay time $t$, thereby constituting the EOC signal ${S}_{{\rm{EOC}}}(t)$.

To demonstrate the broad applicability of our approach, we implement the cost-efficient and widely-available crystalline z-cut α-quartz as the cavity medium, which exhibits a relatively weak EO activity^34^. The extremely broadband, single-cycle THz pulse (Fig. 1b) which transmits into the cavity will undergo numerous internal reflections, leading to the observed pulse-train signal ${S}_{{\rm{EOC}}}(t)$ in Fig. 1c. This intra-cavity signal, while largely corresponding to the cavity field, nevertheless exhibits clear signatures of probing effects. Most notable are the probe-pulse reflections inside the EOC, evidenced by the clear rise-time of the EOC signal’s envelope. To extract the true cavity electric field, we develop a cavity correction function^34–36^ to de-convolve the field from the effects of the probing mechanism (see Methods M2, Fig. S10). Based on EO linear response theory^34,35^, we apply our complex-valued, frequency-domain cavity correction function, ${h}_{{\rm{EOC}}}\left({\varOmega }_{{\rm{THz}}}\right)$, to the EOC spectrum via ${E}_{{\rm{cav}}}\left({\varOmega }_{{\rm{THz}}}\right)={S}_{{\rm{EOC}}}({\varOmega }_{{\rm{THz}}})/{h}_{{\rm{EOC}}}\left({\varOmega }_{{\rm{THz}}}\right)$. This correction function compensates for various probing effects, namely the refractive index mismatch of the THz and visible probe pulses, the cavity reflections of the probe pulse, and the dispersion of the 2nd order nonlinear susceptibility. In contrast to typical detector response functions, we include the reflection of the probe pulses from the gold films, which lead to the prominent rise-time of ${S}_{{\rm{EOC}}}(t)$. By application of this cavity correction function, we obtain the cavity electric field (Fig. 1d), which is the inverse-Fourier transform of the complex, de-convolved spectrum, ${E}_{{\rm{cav}}}\left({\varOmega }_{{\rm{THz}}}\right)$ (Fig. 1e). Notably, the time-domain electric field no longer displays a rise-time, as the probe pulse reflection effects have been de-convolved from the THz electric field. The cavity spectrum reveals clearly-identified cavity modes (red), with a ~$\pi$ phase shift (black) across each cavity resonance, in correspondence with theoretical expectations from periodic sampling of an internally-reflected pulse (see Supplementary Discussion 2). Thus, we have established reliable extraction of quantitative, phase-resolved intra-cavity electric fields, using a simple cavity correction function—a critical step towards characterizing and controlling field-driven phenomena in more complex cavities.

### Monolithic electro-optic cavities

**Fig. 2:**
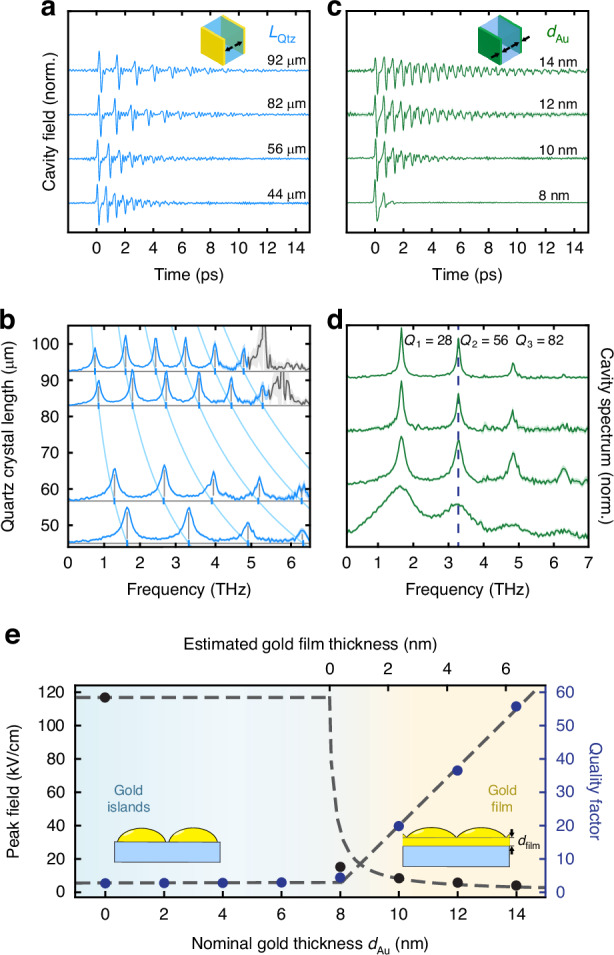
Monolithic electro-optic cavity design. **a** Measured time-domain intra-cavity fields, for quartz cavity lengths of 92, 82, 56, and 44 µm. Nominal deposited gold thickness for all samples is 10 nm. Standard error is represented here, and in panels (**b**–**d**) as a lighter area. **b** Cavity spectra corresponding to the cavity fields in panel (**a**) offset according to the cavity length. The intersections (blue ticks) of the computed cavity dispersions (light blue) with the experimental baselines (horizontal gray lines) highlight the perfect agreement to the experimental mode peaks (vertical gray lines). The gray regions are excluded to suppress zero-crossings of the cavity correction function. (**c**) Measured time-domain intra-cavity fields of a 44 µm-long quartz cavity for nominal deposited gold thicknesses of 14, 12, 10, 8 nm. **d** Cavity spectra corresponding to the fields in panel (**c**) including the mode-specific quality factors for the 14 nm Au mirror cavity (top trace). **e** Intra-cavity peak fields (black dots) and quality factors (blue dots) of the 3.3 THz mode (dashed line in (**d**)), as a function of the gold layer thickness. The dashed lines are derived from electromagnetic modeling

To engineer cavity fields on demand, we precisely design cavity mirror reflectivities and cavity optical lengths by changing the deposited gold film thickness and quartz crystal length, respectively. In Fig. 2a, b, we show the intra-cavity fields, and corresponding spectra, as a function of quartz thickness, thereby adjusting the round-trip time of the THz pulses in the cavity, and therefore the cavity eigenmode spacing. The cavity spectra in Fig. 2b are offset according to the quartz crystal length, demonstrating perfect agreement with the numerically-calculated cavity eigenfrequencies (light blue lines). To illustrate the capability to systematically tailor cavity quality factors, we show in Fig. 2c, d the cavity fields and corresponding spectra as a function of increasing gold film thickness. The cavity fields exhibit an exponential increase in the number of detectable THz pulse internal reflections, and an equivalent linear reduction in the Lorentzian linewidth (see Methods M3). Figure 2e shows the peak fields and quality factors of the 3.3 THz mode (dashed line in Fig. 2d), for a systematic study of deposited gold thicknesses. The delayed onset of cavity signatures is attributed to the nucleation of gold islands during the thin-film growth process. These islands must grow large enough to physically overlap for the emergence of macroscopic metallic behavior to occur^37,38^. The experimental data (dots) are in good agreement with our electromagnetic modeling (dashed lines), with which we have experimentally identified metallic behavior onset at ${d}_{{\rm{Au}}}\approx$ 7.7 nm (see Methods M3 and Fig. S11). This systematic study demonstrates that the cavity quality factor can be tuned to match any fundamental resonance in the THz spectral region and highlights the capability for frequency-tailored EOCs.

### Tunable hybrid electro-optic cavities

**Fig. 3:**
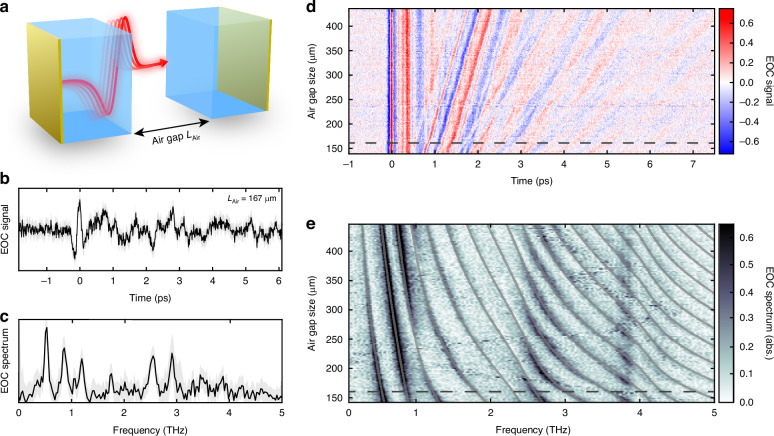
Tunable hybrid electro-optic cavities. **a** Tunable hybrid EOC design, implemented by a pair of electro-optic crystals with gold mirrors on the exterior facets, and a remotely-controllable air gap of size ${L}_{{\rm{Air}}}$. **b** Representative EO signal of a hybrid EOC with an air gap size of ${L}_{{\rm{Air}}}$ = 167 µm. Standard error of EOC signal is represented here and in (**c**) by light areas. **c** Corresponding EOC spectrum of (**b**). **d** Systematic measurement of EOC signal (false color) as a function of the air gap size ${L}_{{\rm{Air}}}$, where the dashed line indicates the gap size shown in (**b**, **c**). **e** Continuous EOC spectra (false color), derived from panel (**d**) with mode eigenvalues from Scattering Matrix Method (gray lines) overlaid

Advancing the concept of frequency-tunable EOCs further, we develop an experimental platform of hybrid EOCs, which allow continuous tuning of the cavity mode frequencies in a single device, while still maintaining the capability for intra-cavity EO sampling. We achieve this, as depicted in Fig. 3a, with a pair of quartz crystals (${L}_{{\rm{Qtz}}}$ = 44 µm), each coated with a gold layer on the exterior facet (${d}_{{\rm{Au}}}$ = 8 nm) and separated by a piezo motor-controllable air gap of size ${L}_{{\rm{Air}}}$. A representative hybrid EOC signal (${L}_{{\rm{Air}}}$ = 167 µm), shown in Fig. 3b, appears evidently more complicated than those observed for the monolithic cavities shown in Fig. 2. The origin of this complexity is revealed in the accompanying spectrum (Fig. 3c), which exhibits numerous cavity modes, with non-equidistant mode frequencies, each with differing signal strengths and linewidths. A systematic scan of the air gap lengths in Fig. 3d, e unveils continuous evolution of discernable features in the time and frequency domains, respectively. We highlight the avoided crossing signatures in Fig. 3e (see details in Fig. S15c), which typically appear in the presence of strong light-matter coupling, as well as an apparent oscillation of mode strength across the observable frequency range.

To better understand these experimental features, we simulate using the Scattering Matrix Method (SMM**;** see Methods M4) the frequency-resolved transmission of the cavity structure, and can thereafter extract the cavity resonance frequencies, which we overlay onto the experimental spectra in Fig. 3e. The perfect agreement of these extracted mode dispersions with our experimental data implies that the observed intensity modulations must arise as a consequence of the hybrid EOC design. Because the SMM simulation does not retain information on the spatial distribution of the fields, it is not capable of providing further physical insight necessary to understand these experimental signatures. Notable among these signatures are the origin of the non-equidistant frequency spacing of the modes, and their periodic signal modulation along the horizontal frequency axis in Fig. 3e, both necessitating further analysis.

**Fig. 4:**
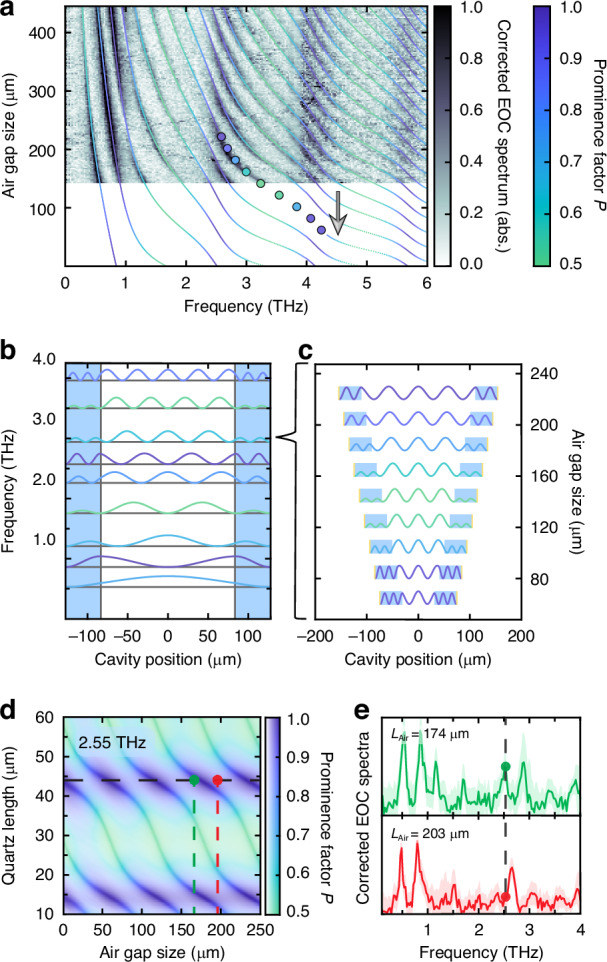
Theoretical modeling of measured hybrid electro-optic cavity features and active EOC design principles. **a** Corrected EOC spectra are displayed (false color), with overlaid eigenvalues obtained from our cavity-field model, color-coded (green→purple) according to their respective prominence factor value ${P}^{q}\left({L}_{{\rm{Air}}}\right)$. **b** Spatial mode intensity profiles for gap size ${L}_{{\rm{Air}}}$ = 167 µm, offset by mode frequency, colored according to the prominence factor (green→purple). The blue shaded regions denotes the quartz layers. **c** Evolution of the 7th cavity mode’s intensity profile plotted as a function of air gap length, displaying the spatial origin of the prominence factor (green→purple) oscillation. **d** The evolution of signal strength (saturation), linewidth, and prominence factor (hue) are shown at 2.55 THz, as a function of quartz length and air gap size. The experimentally-implemented quartz length is denoted by the dashed line. **e** Corrected EOC spectra at air gap sizes corresponding to a resonance at 2.55 THz (top, green), and anti-resonance (bottom, red), corresponding to those in (**d**)

We therefore develop two complementary models—a cavity field-based and a coupled-oscillator model, to understand the signal modulations and the origin of the avoided crossings, respectively. In addition, the cavity-field model^20^ provides a quantitative handle to maximize light-matter coupling at the air-quartz interface where samples can be hosted in future studies. To construct this model, we simply identify cavity electric field solutions which are continuous, and whose wavevectors in air and quartz are dictated by the refractive indices (see Methods M7, and Fig. S12). These few simple assumptions yield: (1) the numerically-calculated eigenfrequencies, ${\varOmega }^{q}\left({L}_{{\rm{Air}}}\right)$ for each mode index q, for every choice of air gap size ${L}_{{\rm{Air}}}$, and (2) for each mode the corresponding spatial field distribution ${E}^{{\rm{q}}}\left({L}_{{\rm{Air}}}{;z}\right)$. To demonstrate the excellent agreement with the experiment, we overlay the eigenvalues obtained from the cavity-field model onto the nonlinear-susceptibility-corrected experimental EOC spectra in Fig. 4a (see Methods M5). For the coupled-oscillator model, however, we consider the cavity as consisting of interacting sub-cavities: the standing wave modes supported natively in the quartz crystals and in the air gap (see Methods M9 and Figs. S15 and S16). The resulting coupled modes not only perfectly reproduce again the experimental eigenvalues, but also provide an intuitive understanding of the origin of the avoided crossing features. In the following, we will show how, taken together, these two models provide comprehensive yet intuitive explanations for the experimental signatures.

We identify that the periodic modulation of the EO signal strength emerges as a consequence of the changes in the modes’ spatial field distributions across the EOC dispersion. Using the analytical field distributions, we quantify the amplitude coefficient of the field within the quartz layers, normalized against the value inside the air gap (see Methods M8). Because this parameter directly reflects the amplitude of the mode inside the quartz crystals where we measure the EO signal, we term this ratio the prominence factor ${P}^{q}$. By coloring the overlaid eigenfrequencies according to their associated prominence factors in Fig. 4a, we highlight the origin of the strong signal modulation arising due to the field suppression within the quartz layers. To further elucidate this relationship, we show in Fig. 4b the calculated spatial structure of the eigenmodes’ intensities, up to 4 THz, for ${L}_{{\rm{Air}}}$ = 167 µm, where each trace is colored according to the prominence factor. Focusing on the 7^th^ eigenmode’s exemplary intensity profile in Fig. 4c, we track its evolution with increasing air gap sizes across one period of the prominence factor cycle, for specific cavity configurations denoted by the dots in Fig. 4a. Strikingly, the prominence factor is maximized (${P}_{\max }$ = 1) when the eigenmode has an anti-node at the air-quartz interface, and minimized (${P}_{\min }={1/n}_{{\rm{Qtz}}}\approx$ 0.5) when there is a node at the interface. We will demonstrate shortly how this connection between the prominence factor and local field amplitudes will be important for purposes of engineered light-matter interactions.

Beyond the signal amplitude modulation, the cavity-field model also reproduces the EOC dispersion, and avoided-crossing signatures therein. It does not, however, provide an intuitive explanation as to their origin. In the coupled-oscillator model, by contrast, the avoided crossings directly emerge from the coupling between standing-wave resonances within the quartz crystals and those in the air gap (see Methods M9, and Figs. S15 and S16). In this model, the coupling strength between the two families of resonances is given roughly by the Fresnel transmission coefficient between the quartz and air layers (see Methods M9 and M10) and is inversely proportional to the THz frequency. Because both models reproduce the EOC dispersion perfectly, the regions of high prominence factor are correlated with eigenmodes possessing a large quartz standing-wave character. This eigenmode character has implications not only for EOC field sensing, but also for future studies of light-matter interactions, as we explore next.

Precise and targeted cavity design will play an important role for future studies of intra-cavity sampling of strong light-matter coupling. As a first step, we demonstrate how the prominence factor should be consulted as a design parameter for light-matter interaction, in addition to its role in EOC-sampling sensitivity presented so far. The prominence factor ${P}^{q}$ is directly proportional to the electric field at the air quartz interface, according to ${E}_{\mathrm{int}}\propto ({P}^{q}-1/{n}_{{\rm{Qtz}}})/(1-1/{n}_{{\rm{Qtz}}})$. Therefore, a thin sample placed or grown on the interior electro-optic crystal facet will experience that same relative interfacial field ${E}_{\mathrm{int}}$. Because the relative light-matter interaction strength scales quadratically with respect to this interfacial field^20^, tailoring the frequency-dependent prominence factor ${P}^{q}$ plays a key role. We depict in Fig. 4d the modeled evolution of the prominence factor and the theoretical signal strength (see Methods M6) as a function of quartz crystal length and air gap size, for an exemplary target frequency of 2.55 THz, for which the prominence factor of the implemented hybrid EOC is already maximal. With this, we experimentally show in Fig. 4e that our hybrid EOC can be actively tuned, from resonant to anti-resonant at a specific design frequency, demonstrating the capability for switchable light-matter coupling that can be directly investigated in an appropriately-tailored EOC. We thus have shown that the cavity-field and coupled-oscillator models described herein provide not only understanding for the measured EOC signal features, but additionally offer critical cavity-design principles for future in-situ studies of tunable light-matter coupling.

## Discussion

Our results establish in-situ cavity field measurement in EOCs as a new, integrated probe for low-energy cavity electrodynamics. Time-domain measurements of cavity fields are more direct than conventional intensity-based spectroscopy, where mode details are only implicitly described from linewidths and without phase information^1,39^. Extending field-resolved methods to include intra-cavity sampling now unlocks the additional advantages of enhanced, quantitative measurement of the local cavity fields, in the absence of distortion due to either reflections in cavity substrates, or end-mirror dispersion (see Fig. S11). In particular, the reduction in probe field strength due to the cavity reflectivity is very small compared to the relative enhancement of the principal THz pulse peak field strengths inside the cavity, as compared to the transmitted THz pulse, which is quantified by $1/{t}_{\mathrm{int}}^{{\rm{THz}}}$ (see Methods M3, Figs. S11a, b). We experimentally confirm this enhancement by comparison to the transmitted field (see Supplementary Discussion 5 and Fig. S7), whereby we observe a six-fold enhancement of the cavity field strength for a nominal gold thickness of 10 nm. We therefore estimate a relative THz-field enhancement of 25 for the highest quality cavity (${d}_{{Au}}\,$ = 14 nm) investigated here.

Our hybrid EOC design extends these concepts by providing continuous tunability and the potential for adding active samples for investigations of intra-cavity light-matter interactions. In the ‘empty’ hybrid cavity investigated here, we observe a rich mode structure, spurring development of both a field-based model to quantify these cavity modes and their properties, as well as a complementary coupled-oscillator description to gain further understanding of the delicate interplay between the various sub-cavities, which thereafter constitute the hybrid EOC modes. Our detailed analysis of these theoretical vantage points will be highly valuable when considering the addition of an active material, after which the hybrid cavity optical response will become even more intricate. Integration of active materials into hybrid EOCs will yield novel access to light-matter interactions—namely access to energy exchange on sub-Rabi-cycle timescales, and furthermore local probing and even control over tunable light-matter superposition—the latter two unavailable when viewed by conventional cavity transmission techniques. Potential ‘active materials’ for these in-situ investigations of tunable light-matter interactions include conventional polar semiconductors^40^—oftentimes displaying very large oscillator strengths—atomically-thin monolayers or heterostructures of transition-metal dichalcogenides^41^, hybrid organic-inorganic 3D^21,42^ and 2D lead-halide perovskites^43,44^, and novel, magnetically-ordered systems^45^.

Implementation of EO sampling inside of THz cavities will also significantly advance further areas of contemporary research. As a prominent example, field-resolved probing inside a defined electromagnetic cavity will provide novel opportunities for measurements of electromagnetic vacuum field fluctuations^46,47^. Most notably, a high-quality factor EOC constitutes an advantageous testing ground for measurement of quantum vacuum fluctuations, by efficiently excluding sources of external radiation. Moreover, EOCs are not limited to either macroscopic environments or the THz spectral region. Although EO sampling is routinely employed up to the mid-IR spectral region^9^, it has recently been extended even into the visible range^48^, allowing for future broadband measurements of intra-cavity electric fields. Similar sampling techniques have been used to sample electric fields inside of metallic antenna-based cavities^49,50^, demonstrating that although on-chip photonic implementations lack the dynamic tunability, the general technique is readily implemented in other near-field contexts, including even tip-based nano-photonic applications^51^. Furthermore, EOCs utilizing quartz are uniquely suited candidates for chiral THz cavity phenomena^52^, due to quartz’s capability for straightforward and rapid measurement of vectorial electric field trajectories^34^.

In conclusion, we have established versatile and compact designs for a new class of active THz cavities, which allow for in-situ retrieval of intra-cavity electric fields. By developing a cavity-correction function formalism for these EOCs, we have demonstrated a rigorous and reliable method to extract absolute fields in a quantitative, and phase-resolved manner. Utilizing straightforward fabrication techniques, we tune the cavities’ quality factors and resonance frequencies. Furthermore, we have introduced a hybrid EOC, offering continuously-tunable cavity modes across the entire THz-frequency range, within a single device. This fundamental advancement lays the groundwork for accommodating additional active materials for in-situ measurement of and control over light-matter coupling. We understand the rich hybrid mode structure, including apparent signatures of strong coupling, via cavity-field and coupled-oscillator formalisms, which will be key to deciphering signatures of light-matter coupling in more complicated devices. Therefore, this work opens new dimensions of THz cavity physics, particularly in the realms of cavity-controlled ground- and excited state material properties. This includes possibilities such as cavity-enhanced THz emission, selectively-driven Floquet states^53^, and cavity-controlled nonlinear THz driving^15,54^, thus paving the way for comprehensive investigations of THz cavity quantum electrodynamics.

## Materials and methods

### (M1) Materials and experimental setup

We performed all electro-optic sampling measurements using high-field THz pulses emitted from a large-area spintronic emitter^10^, where the pulses were generated by 1 kHz, 2.5 mJ, 796 nm laser pulses, with Fourier-limited pulse durations of 38 fs. The THz pulses at this pump power have maximum field strengths of ~200 kV/cm, as measured by EO sampling, and are extremely broadband (maximum spectral strength at 2.0 THz, with a FWHM bandwidth of ~3.5 THz), and temporally short (electric field FWHM of $\tau$ ≅ 100 fs). These THz pulses are sampled using 20-fs pulse duration, 400 pJ, 791 nm broadband laser pulses from a Vitara oscillator.

Cavity electro-optic sampling measurements are performed using a heterodyne detection scheme, where both the transmitted probe pulse and the pulse emitted from the nonlinear polarization are detected simultaneously on a frequency-insensitive photodiode pair. The sum of these two pulses can be treated as a single probe pulse which has experienced an effective field-induced birefringence^35^, which is experimentally extracted using a balanced-detection scheme (see Fig. S9). This induced rotation is translated into a quantitative cavity electric field using the numerical cavity correction function that is described below.

The gold films were deposited using the standard technique of electron-beam physical vapor deposition. The depositions were performed under ultra-high vacuum conditions of ~7 picobars. The nominal deposited gold thicknesses reported herein were monitored via a quartz crystal microbalance.

### (M2) Cavity correction function

The functional form of the cavity correction function used to analyze the EOC signal (see Supplementary Discussion 3 for extended discussion) is^34^^,35,55^:

M.1 $$\begin{array}{c}{h}_{{\rm{fun}}}\left({\varOmega }_{{\rm{THz}}}\right)={\chi }_{{\rm{eff}}}^{\left(2\right)}\left({\varOmega }_{{\rm{THz}}}\right)\int \frac{{\omega }_{{\rm{vis}}}^{2}}{{{{c}}}_{{\rm{o}}}^{2}{\rm{k}}\left({\omega }_{{\rm{vis}}}\right)}{T}_{\rm{pr}}\left({\omega }_{{\rm{vis}}},{\varOmega }_{{\rm{THz}}}\right){{E}}_{{\rm{pr}}}^{* }\left({\omega }_{{\rm{vis}}}\right)\cdots \\ {{E}}_{{\rm{pr}}}\left({\omega }_{{\rm{vis}}}-{\varOmega }_{{\rm{THz}}}\right)G\left({\omega }_{{\rm{vis}}},{\varOmega }_{{\rm{THz}}}\right){\rm{d}}{\omega }_{{\rm{vis}}}\end{array}$$

Here, ${{E}}_{{\rm{pr}}}\left({\omega }_{{\rm{vis}}}\right)$ is the spectrum of the incident visible-frequency probing pulse, and ${{\rm{E}}}_{{\rm{pr}}}\left({\omega }_{{\rm{vis}}}-{\varOmega }_{{\rm{THz}}}\right)$ that of the frequency-shifted pulse emitted from the nonlinear polarization, for a given THz frequency. The other terms ${T}_{{\rm{pr}}}$, ${\chi }_{{\rm{eff}}}^{\left(2\right)}$, and $G$ will be discussed in detail shortly. In the assumption of no dispersion within the bandwidth of the probe spectrum, i.e. by assuming that for the quantitative, nonlinear susceptibility ${\chi }_{{\rm{eff}}}^{\left(2\right)}\left({\varOmega }_{{\rm{THz}}},{\omega }_{{\rm{vis}}}\right)\approx {{{\chi }}}_{{\rm{eff}}}^{\left(2\right)}\left({\varOmega }_{{\rm{THz}}}\right)$, the sum-frequency and difference-frequency generation signals differ only in terms of phase, and therefore the total quantitative response function applied to the experimental data is thus given by:

M.2 $$h\left({\varOmega }_{{\rm{THz}}}\right)=\frac{{h}_{{\rm{fun}}}\left({\varOmega }_{{\rm{THz}}}\right)+{h}_{{\rm{fun}}}^{* }\left({-\varOmega }_{{\rm{THz}}}\right)}{\int {{E}}_{{\rm{pr}}}\left({\omega }_{{\rm{vis}}}\right){{E}}_{{\rm{pr}}}^{* }\left({\omega }_{{\rm{vis}}}\right){\rm{d}}{\omega }_{{\rm{vis}}}}$$

This cavity correction function $h\left({\varOmega }_{{\rm{THz}}}\right)$ accounts for numerous non-local probing effects, each displayed in Fig. S10, and discussed next.

The phase mismatch function ${{G}}\left({\omega }_{{\rm{vis}}},{\varOmega }_{{\rm{THz}}}\right)$ (Fig. S10a) accounts for the accumulated phase difference between the co-propagating THz and probing fields, and is given by:

M.3 $$G\left({\omega }_{{\rm{vis}}},{\varOmega }_{{\rm{THz}}};{{L}}_{{\rm{Qtz}}}\right)=\frac{{{\rm{e}}}^{{i}\Delta {k}_{{\rm{co}}}\left({\varOmega }_{{\rm{THz}}},{\omega }_{{\rm{vis}}}\right){{L}}_{{\rm{Qtz}}}}-1}{{i}\Delta {k}_{{\rm{co}}}\left({\varOmega }_{{\rm{THz}}},{\omega }_{{\rm{vis}}}\right)}$$

This phase mismatch depends on the fundamental momentum mismatch of the co-propagating THz and visible waves $\Delta {k}_{{\rm{co}}}\left({\varOmega }_{{\rm{THz}}},{\omega }_{{\rm{vis}}}\right)\approx \left({{{n}}}_{{\rm{THz}}}\left({\varOmega }_{{\rm{THz}}}\right)-{{{n}}}_{{\rm{vis}}}\right){\varOmega }_{{\rm{THz}}}$, where we have assumed the refractive index in the spectral region of the visible probe pulse is practically dispersionless.

The frequency-dependent nonlinear susceptibility (Fig. S10b) is given by:

M.4 $${\chi }_{{\rm{eff}}}^{\left(2\right)}\left({\varOmega }_{{\rm{THz}}}\right)={\chi }_{{\rm{e}}}^{\left(2\right)}\left[1+\sum _{j}\frac{{C}_{j}{\varOmega }_{j}^{2}}{{\varOmega }_{j}^{2}-{\varOmega }_{{\rm{THz}}}^{2}-i{\varOmega }_{{\rm{THz}}}{\varGamma }_{j}}\right]$$

Here, ${\chi }_{{\rm{e}}}^{\left(2\right)}$ is the purely-electronic (i.e. non-resonant) component of the 2^nd^-order susceptibility, whose numerical value is taken from Frenzel, et al.^34^. The remaining sum represents the resonant ionic terms, written with amplitudes relative to the electronic term. The effective nonlinear susceptibility parameters used here are tabulated for reference in SI Table 2.

The probe pulse transmission ${T}_{{\rm{pr}}}\left({\omega }_{{\rm{vis}}},{\varOmega }_{{\rm{THz}}}\right)$ (see Supplementary Discussion 2 and Fig. S10c) is given by the following:

M.5 $${T}_{\rm{pr}}\left({\omega }_{{\rm{vis}}},{\varOmega }_{{\rm{THz}}}\right)=\frac{{t}_{\mathrm{int}}({\omega }_{{\rm{vis}}})}{1-{{{r}}_{\mathrm{int}}^{2}\left({\omega }_{{\rm{vis}}}\right)}{{\rm{e}}}^{{i}{\varOmega }_{{\rm{THz}}}{\tau }_{{\rm{RT}}}\left({\omega }_{{\rm{vis}}}\right)}}$$

This serves to account for both the Fabry-Pérot internal reflections (denominator)—separated in time by the visible-frequency cavity round-trip time ${\tau }_{{\rm{RT}}}=2{n}_{{\rm{Qtz}}}\left({\omega }_{{\rm{vis}}}\right){L}_{{\rm{Qtz}}}/{{\rm{c}}}_{{\rm{o}}}$, where ${{\rm{c}}}_{{\rm{o}}}$ is the vacuum speed of light- and the transmission out of the cavity to reach the photodiode detector is given by ${t}_{\mathrm{int}}({\omega }_{{\rm{vis}}})\approx 1-{r}_{\mathrm{int}}\left({\omega }_{{\rm{vis}}}\right)$. The internal reflectivity ${r}_{\mathrm{int}}$ (defined in Supplementary Discussion 1) is implemented here as a real number, whose correct value is identified by optimal deconvolution with probe cavity reflections, where cavity pulses will otherwise appear (see Fig. 1c) as signals at negative delay times. In contrast to the conventional application of a detector response function, we do not consider the Fresnel coefficients for the transfer of the incident pulses into the cavity, as we do not intend to infer the fields incident to the cavity, but strictly the intra-cavity fields.

Finally, for the purpose of comparison with electromagnetic modeling (see Figs. S11a, b), we also consider the Fabry-Pérot reflections for the THz intra-cavity pulse (Fig. S10d):

M.6 $${T}_{{\rm{THz}}}\left({\varOmega }_{{\rm{THz}}}\right)=\frac{1}{1-{{r}^{2}_{\mathrm{int}}\left({\varOmega }_{{\rm{THz}}}\right)}{{\rm{e}}}^{-{i}{\varOmega }_{{\rm{THz}}}{\tau }_{{\rm{RT}}}\left({\varOmega }_{{\rm{THz}}}\right)}}$$

By including this additional term in the cavity correction function, we identify the principal single-cycle pulse measured inside the cavity (Fig. S10g).

We have considered here only measurement of forward-propagation of the principal THz pulse inside the cavity. In Supplementary Discussion 3 we consider both forwards- and backwards propagation of the principle pulse (see Fig. S5) and characterize our weak measurement of the backwards propagation.

### (M3) Monolithic cavity characterization

The monolithic cavities are characterized by: (1) round trip times, ${{\rm{\tau }}}_{{\rm{RT}}}=2{n}_{{\rm{Qtz}}}{L}_{{\rm{Qtz}}}/{{{c}}}_{{\rm{o}}}$, and (2) by quality factors, ${Q}_{{{q}}}={\varOmega }_{{{q}}}/{\rm{\delta }}{\varOmega }_{{{q}}}$, defined for the *q*^th^ mode as the ratio between the cavity mode’s angular frequency, ${\rm{\omega }}_{q}$, and its linewidth, computed in terms of energy or spectral intensity. There is a common, alternative, definition of the quality factors ${Q}_{{\rm{q}}}$ in terms of the ratio between the mode frequency and the frequency associated with the energy loss rate: ${Q}_{{{q}}}={\varOmega }_{{{q}}}/\left(\frac{{l}_{{\rm{RT}}}}{{{\rm{\tau }}}_{{\rm{RT}}}}\right)$, where ${l}_{{\rm{RT}}}$ is the fractional round-trip energy loss. Refer to Supplementary Discussion 2 for further discussion of the round-trip energy-loss rate. Because the cavity frequencies are, neglecting dispersion, multiples of the inverse round trip time, this secondary expression reduces to ${Q}_{{\rm{q}}}=2{\rm{\pi }}{{q}}/{l}_{{\rm{RT}}}$, where it must however be stressed that this equation is only valid in situations where ${l}_{{\rm{RT}}}\ll 1$.

To display in Fig. 2b the evolution of the cavity eigenfrequencies as a function of the quartz crystal length, the cavity eigenfrequencies must be numerically computed if dispersion from the refractive index is to be accounted for. The refractive index used for numerical simulations here is reported in SI Table 1. To achieve this, we identify the frequency ${f}_{{\rm{THz}}}^{q}={\varOmega }_{{\rm{THz}}}^{q}/2\pi$ for a given mode index q, and quartz length ${L}_{{\rm{Qtz}}}$, which satisfy:

M.7 $$\frac{{\varOmega }_{{\rm{THz}}}^{q}}{2{\rm{\pi }}}-q\left(\frac{{{{c}}}_{{\rm{o}}}}{2{L}_{{\rm{Qtz}}}{n}_{{\rm{Qtz}}}\left({\varOmega }_{{\rm{THz}}}^{q}\right)}\right)=0$$

We also apply in Fig. 2b a masking function, in order to suppress the zero-crossing of the phase mismatch function $G\left({\omega }_{{\rm{vis}}},{\varOmega }_{{\rm{THz}}};{L}_{{\rm{Qtz}}}\right)$ in longer quartz crystals (see Supplementary Discussion 4, and Fig. S6).

In Fig. 2e, we show the correspondence between the experimental and simulated fields and quality factors. The experimental field strengths are extracted from the full cavity correction function, where we additionally make a simple correction for probe scattering from gold island nanostructures^38,56^. In this approximation, we track the probe power as a function of gold film thickness, and correct the cavity probe fields by characterizing the intensity-proportional scattering associated with the gold islands (see Supplementary Discussion 6 and SI Fig. 8). The quality factors are derived from fitting the cavity fields in Fig. 2d, according to ${{Q}}_{{{q}}}={{{\Omega}}}_{{{q}}}/{\rm{\delta}}{{{\Omega}}}_{{{q}}}$. The theoretical results are obtained via computation using a three-medium model, where a distinction is made between fields traveling into the cavity, versus out of the cavity (see Supplementary Discussion 1). The results are shown in Figs. S11a, b, displaying good agreement not only with the information shown in Fig. 2e, but also to the internal THz and visible reflection coefficients inferred from the cavity correction function. In this analysis, we identify best-correspondence Drude model parameters for the gold metal films of $\hslash {\omega }_{{\rm{plasma}}}$ = 8.5 eV, ${\Gamma }_{{\rm{damp}}}$ = 8 THz, where we retain the plasma frequency from Olmon et al.^57^. and varied the damping rate and film onset thickness to identify optimal agreement with the experimentally-measured quality factors and internal cavity peak field strengths.

This three-medium model is then used to compute the resultant principal pulses emitted from an EOC, using the experimentally-identified Drude parameters, to demonstrate the fundamental effects of dispersion caused by the gold film interfaces (Fig. S11c–e). We note that for the relatively thin films used in this study, the effect of spectral dispersion due to the Fresnel coefficients at the gold-quartz interface dominates the pulse dispersion, leading to a suppression of low frequencies in the transmitted pulses. Higher quality cavities, which may be probed with more sensitive electro-optic crystals, will eventually suffer from absorptive effects in thicker gold films, leading to the opposite effect.

### (M4) Numerical simulation—scattering matrix method

To simulate the total field transmission of the hybrid EOC, we implement a numerical solution of Maxwell’s Equations called the Scattering Matrix Method (SMM), a variant of the Transfer Matrix Method (TMM), optimized for the case of a strictly isotropic system^58^. TMM propagates the fields through a one-dimensional, layered electromagnetic structure, taking into account the boundary conditions at every interface via a transfer function, and the phase and absorption accumulated across each region. SMM, by contrast, uses a global scattering matrix which is constructed across the entire structure, and used to relate input fields to output fields. This is a more memory-efficient method, and is constructed strictly for linear, homogeneous, and isotropic materials. This efficiency comes at the cost of an inherent lack of spatially-resolved information, due to the formulation of the utilized global scattering matrix.

### (M5) Hybrid cavity—corrected EOC spectrum

The ‘corrected’ EOC spectra presented in Fig. 4a, e are obtained by taking the absolute value of the experimental EOC spectra after dividing by the frequency-dependent, second-order, effective susceptibility ${\chi }_{{\rm{eff}}}^{\left(2\right)}\left({\varOmega }_{{\rm{THz}}}\right)$, both of which are themselves complex quantities.

M.8 $${S}_{{\rm{EOC}}}^{{\rm{corr}}}\left({\varOmega }_{{\rm{THz}}}\right)=\left|\frac{{S}_{{\rm{EOC}}}\left({\varOmega }_{{\rm{THz}}}\right)}{{\chi }_{{\rm{eff}}}^{\left(2\right)}\left({\varOmega }_{{\rm{THz}}}\right)}\right|$$

In this way, we suppress the ionic resonance in ${\chi }_{{\rm{eff}}}^{\left(2\right)}\left({\varOmega }_{{\rm{THz}}}\right)$ evident in the raw experimental spectra (see Fig. 3e), in order to highlight the role of the prominence factor in determining the overall signal strength.

### (M6) Hybrid cavity—simulated mode strength

We compute the simulated mode strength $S(\varOmega ;{L}_{{\rm{Air}}},{L}_{{\rm{Qtz}}})$ displayed in Fig. 4d, by constructing a series of complex Lorentzian characteristic lineshapes (see Supplementary Discussion 2), centered at the numerically-identified eigenfrequencies ${\varOmega }_{{\rm{THz}}}^{q}\left({L}_{{\rm{Qtz}}},{L}_{{\rm{Air}}}\right)$, with linewidths $\Gamma ={l}_{{\rm{RT}}}{P}^{q}\left({L}_{{\rm{Qtz}}},{L}_{{\rm{Air}}}\right)/{\tau }_{{\rm{RT}}}$, i.e. the product of the fundamental loss rate with the prominence factor, divided by the round-trip time. Finally, we multiply by the prominence factor associated to each model oscillator, in order to incorporate, to lowest order, the effect of probing the THz field only inside the electro-optic crystal.

M.9 $$S({\varOmega }_{{\rm{THz}}}{;}\,{L}_{{\rm{Air}}},{L}_{{\rm{Qtz}}})=\sum _{q}{P}^{q}\left({L}_{{\rm{Qtz}}},{L}_{{\rm{Air}}}\right)\left|\frac{\frac{\varGamma }{2}}{{i}\left({\varOmega }_{{\rm{THz}}}-{\varOmega }_{{\rm{THz}}}^{q}\left({L}_{{\rm{Qtz}}},{L}_{{\rm{Air}}}\right)\right)+\frac{\varGamma }{2}}\right|$$

### (M7) Hybrid cavity—field-based model

We implement a field-based model to theoretically simulate the cavity eigenfrequencies and eigenmodes that are experimentally observed within the hybrid EOCs. To find these eigenmodes, we look for cavity electric fields which satisfy the following constraints: (1) the field vanishes at the EO crystal-gold boundaries, (2) that the field is continuous across the air-crystal boundaries, and (3) that the first derivative is also continuous across these boundaries. These requirements generate from the initial ansatz of plane waves (see Fig. S12a) two classes of solutions, corresponding to modes having either even ($C=D$) or odd ($D=-C$) parity symmetry, and which have cavity eigenfrequencies identified via numerical solutions to the following transcendental equations:

M.10 $$\frac{{k}_{{\rm{EO}}}}{{k}_{{\rm{o}}}}\cot \left({k}_{{\rm{EO}}}{{{L}}}_{{\rm{EO}}}\right)=\tan \left({k}_{{\rm{o}}}{L}_{{\rm{o}}}\right),\quad \frac{{k}_{{\rm{EO}}}}{{k}_{\rm{o}}}\cot \left({k}_{{\rm{EO}}}{{{L}}}_{{\rm{EO}}}\right)=-\cot \left({k}_{{\rm{o}}}{L}_{{\rm{o}}}\right)$$

where the first equation corresponds to modes possessing even parity symmetry, and the second equation odd ones. In these equations, the wavevector in air is given by ${k}_{\rm{o}}={\varOmega }_{{\rm{o}}}/{c}_{\rm{o}}$, where ${\varOmega }_{\rm{o}}=2\pi {f}_{\rm{o}}$ is the THz angular frequency, and ${{{c}}}_{{\rm{o}}}$ the vacuum speed of light. The wavevector inside of the electro-optic crystal is then ${k}_{{\rm{EO}}}={\varOmega }_{{\rm{o}}}/{{c}}$ where the speed of light in the crystal is given by $c={c}_{\rm{o}}/\sqrt{\left({\epsilon }_{\rm{EO}}\right.\left({\varOmega }_{\rm{o}}\right)}$. Finally, ${{L}}_{{\rm{EO}}}$ is the length of the electro-optic crystal, and ${L}_{\rm{o}}$ is equal to half of the total air gap ${L}_{{\rm{Air}}}$, i.e. ${L}_{{\rm{Cav}}}=2{L}_{{\rm{EO}}}+{L}_{{\rm{Air}}}$. The corresponding electric fields inside the cavity are then determined to be the following:

M.11 $${E}_{{\rm{even}}}\left(z\right)=\left\{\begin{array}{l}\frac{\cos \left({k}_{{\rm{o}}}{L}_{{\rm{o}}}\right)}{\sin \left({k}_{{\rm{EO}}}{L}_{{\rm{EO}}}\right)}\sin \left({k}_{{\rm{EO}}}\left(z+\frac{{L}_{{\rm{Cav}}}}{2}\right)\right),\quad{-L}_{{\rm{o}}}-{L}_{{\rm{EO}}}\le z < -{L}_{{\rm{o}}}\\ \qquad\quad\cos \left({k}_{{\rm{o}}}z\right),\qquad\qquad\qquad\qquad\qquad\left|z\right|\le {L}_{{\rm{o}}}\\ \frac{-\cos \left({k}_{{\rm{o}}}{L}_{{\rm{o}}}\right)}{\sin \left({k}_{{\rm{EO}}}{L}_{{\rm{EO}}}\right)}\sin \left({k}_{{\rm{EO}}}\left(z-\frac{{L}_{{\rm{Cav}}}}{2}\right)\right),\quad{L}_{{\rm{o}}}> z \ge {L}_{{\rm{o}}}+{L}_{{{EO}}}\end{array}\right.$$

M.12 $${E}_{{\rm{odd}}}\left(z\right)=\left\{\begin{array}{l}\frac{-{i}\sin \left({k}_{{\rm{o}}}{L}_{{\rm{o}}}\right)}{\sin \left({k}_{{\rm{EO}}}{L}_{{\rm{EO}}}\right)}\sin \left({k}_{{\rm{EO}}}\left(z+\frac{{L}_{{\rm{Cav}}}}{2}\right)\right), \quad {-L}_{{\rm{o}}}-{L}_{{\rm{EO}}}\le z < -{L}_{{\rm{o}}}\\ \qquad\quad{i}\sin \left({k}_{{\rm{o}}}z\right),\qquad\qquad\qquad\qquad\qquad\left|z\right|\le {L}_{{\rm{o}}}\\ \frac{-{i}\sin \left({k}_{{\rm{o}}}{L}_{{\rm{o}}}\right)}{\sin \left({k}_{{\rm{EO}}}{L}_{{\rm{EO}}}\right)}\sin \left({k}_{{\rm{EO}}}\left(z-\frac{{L}_{{\rm{Cav}}}}{2}\right)\right), \quad {L}_{{\rm{o}}}> z \ge {L}_{{\rm{o}}}+{L}_{{\rm{EO}}}\end{array}\right.$$

Further details on obtaining these expressions can be found in Supplementary Discussion 7. The fields for a select few modes are presented in Fig. S12d, depicting the effect of air gap-size tuning, and the associated prominence factor, as discussed in the next section.

Additionally, we use Snell’s law to account for refraction at the air-crystal interfaces, where the incidence angle, ${\theta }_{{\rm{inc}}}$, and transmitted angle, ${\theta }_{{\rm{tr}}},$ are defined in terms of the incident wavevector:

M.13 $$\sin \left({{{\theta }}}_{{\rm{inc}}}\right)=\frac{{k}_{\perp }}{{k}_{{\rm{inc}}}}\,\sin \left({{{\theta }}}_{{\rm{inc}}}\right)={{n}}_{{\rm{EO}}}\sin \left({{{\theta }}}_{{\rm{tr}}}\right)$$

Using this, we generalize the field-based model to non-normal angles of incidence, resulting in the updated transcendental equations:

M.14 $$\frac{{k}_{{\rm{EO}}}}{{k}_{{\rm{o}}}}\cot \left({k}_{{\rm{EO}}}\cos \left({\theta }_{{\rm{tr}}}\right){{{L}}}_{{\rm{EO}}}\right)=\tan \left({k}_{\rm{o}}\cos{({\theta }_{{\rm{inc}}})}{L}_{{\rm{o}}}\right)$$

M.15 $$\frac{{k}_{{\rm{EO}}}}{{k}_{{\rm{o}}}}\cot \left({k}_{{\rm{EO}}}\cos \left({\theta }_{{\rm{tr}}}\right){{{L}}}_{{\rm{EO}}}\right)=-\cot \left({k}_{{\rm{o}}}\cos \left({\theta }_{{\rm{inc}}}\right){L}_{{\rm{o}}}\right)$$

We demonstrate in Fig. S13 the momentum-dependent dispersion of a representative hybrid cavity’s modes, demonstrating why we neglect effects of cavity dispersion in our analysis of hybrid EOC field measurements.

### (M8) Hybrid cavity—prominence factor

We define the prominence factor as the absolute value of the normalization coefficient in the EO crystal layer, as identified from the cavity field model:

M.16 $$\begin{array}{lll}{P}_{{\rm{even}}}^{q}\left({L}_{{\rm{EO}}},{L}_{{\rm{Air}}}\right)=\left|\frac{\cos \left({k}_{\rm{o}}{L}_{\rm{o}}\right)}{\sin \left({k}_{{\rm{EO}}}{L}_{{\rm{EO}}}\right)}\right|\\\,\,{P}_{{\rm{odd}}}^{q}\left({L}_{{\rm{EO}}},{L}_{{\rm{Air}}}\right)=\left|\frac{\sin \left({k}_{\rm{o}}{L}_{\rm{o}}\right)}{\sin \left({k}_{{\rm{EO}}}{L}_{{\rm{EO}}}\right)}\right|\end{array}$$

The prominence factor has a detailed evolution for each mode, according to its dispersion (Figs. S12b, d). However, when evaluated as a function of frequency, we see that all modes’ prominence factors lie on a single curve (see Fig. S12c). We identify the functional form of this curve as the following:

M.17 $$P\left({\varOmega }_{{\rm{THz}}}{;}\,{L}_{{\rm{EO}}}\right)=\left|\frac{{{{t}}}_{{\rm{EO}}}^{\mathrm{int}}\left({\varOmega }_{{\rm{THz}}}\right)}{1+{\left({{{r}}}_{{\rm{EO}}}^{\mathrm{int}}\left({\varOmega }_{{\rm{THz}}}\right)\right)}^{2}{{\rm{e}}}^{-{i}{\varOmega }_{{\rm{THz}}}{\tau }_{{\rm{RT}}}\left({L}_{{\rm{EO}}}\right)}}\right|$$

Here we have used ${{r}}_{{\rm{EO}}}^{\mathrm{int}}=({{n}}_{{\rm{EO}}}-1)/({{n}}_{{\rm{EO}}}+1)$, and ${{t}}_{{\rm{EO}}}^{\mathrm{int}}=1-{{r}}_{{\rm{EO}}}^{\mathrm{int}}$ the internal reflection and transmission coefficient from the EO crystal into air, and ${\tau }_{{\rm{RT}}}\left({L}_{{\rm{EO}}}\right)=2{{n}}_{{\rm{EO}}}{L}_{{\rm{EO}}}/{{{c}}}_{{\rm{o}}}$. This function oscillates between the value of $1$ and $1/{{n}}_{{\rm{EO}}}$ (see Fig. S16c) and can be understood as an analog to the Fabry-Pérot transfer functions considered in the cavity correction function, except where the constructive interference conditions are fundamentally modified by the gold mirror, as discussed next.

### (M9) Hybrid cavity—coupled-oscillator model

With this model, we demonstrate that the measured cavity eigenfrequencies may be understood in terms of the interaction of simple standing waves that are sustained in the individual components that constitute the total cavity structure (Figs. S14–17). We consider coupling between standing wave resonances in both the electro-optic crystals and the air gap separating them. In the air gap, the modes have path lengths of half-integer multiples of the wavelength, corresponding to typical Fabry-Pérot standing waves (resonances). The modes in the EO crystals have optical path lengths of odd-integer multiples of a quarter of the wavelength in the crystal, differing from those in the air gap as a consequence of the nodal point imposed by the external gold mirror (Fig. S15a). The cavity THz frequencies for these uncoupled modes are therefore given by:

M.18 $${f}_{{\rm{EO}}}^{t}=\frac{{\varOmega }_{{\rm{EO}}}^{t}}{2\pi }=\frac{2t-1}{4}\frac{{{{c}}}_{{\rm{o}}}}{{L}_{{\rm{EO}}}{{n}}_{{\rm{EO}}}\left({f}_{{\rm{EO}}}^{t}\right)},\quad t\in \left\{-{{\infty }},\ldots -1,1,\ldots ,{{\infty }}\right\}$$

M.19 $${f}_{{\rm{Air}}}^{u}=\frac{{\varOmega }_{{\rm{Air}}}^{u}}{2\pi }=\frac{u}{2}\frac{{{{c}}}_{{\rm{o}}}}{{L}_{{\rm{Air}}}}, \quad u\in \left\{-{{\infty }},\ldots -1,0,1,\ldots ,{{\infty }}\right\}$$

For a generic value of THz frequency $f$, we consider a finite number ${{\rm{N}}}_{{\rm{EO}}}$ of possible resonance conditions in the EO crystals and ${{\rm{N}}}_{{\rm{Air}}}$ possible resonance conditions in the air gap. These correspond to discrete cavity configurations, i.e. values of electro-optic crystal length ${L}_{{\rm{EO}}}$ and air gap size ${L}_{{\rm{Air}}}$, respectively, which fulfill:

M.20 $${{n}}_{{\rm{EO}}}\left(f\right){L}_{{\rm{EO}}}^{t}\left(f\right)=\frac{2t-1}{4}\frac{{{{c}}}_{{\rm{o}}}}{f},\quad t\in \left\{-{{\rm{N}}}_{{\rm{EO}}},\ldots -1,1,\ldots ,{{\rm{N}}}_{{\rm{EO}}}\right\}\equiv T$$

M.21 $${L}_{{\rm{Air}}}^{u}\left(f\right)=\frac{u}{2}\frac{{{{c}}}_{{\rm{o}}}}{f}, \quad u\in \left\{-{{\rm{N}}}_{{\rm{Air}}},\ldots -1,0,1,\ldots ,{{\rm{N}}}_{{\rm{Air}}}\right\}\equiv U$$

Normalizing these expressions by the free-space wavelength ${\lambda }_{{\rm{o}}}={c}_{{\rm{o}}}/f$ yields the following series of equations of lines (see Fig. S15b, d):

M.22 $${{\mathcal{M}}}_{{\rm{EO}}}^{t}=\frac{2t-1}{4},\quad {{\mathcal{M}}}_{{\rm{Air}}}^{u}=\frac{u}{2}$$

We identify these quantities generally as the number of optical cycles in the electro-optic medium ${{\mathcal{M}}}_{{\rm{EO}}}^{t}={n}_{{\rm{EO}}}\left(f\right){L}_{{\rm{EO}}}^{t}\left(f\right)f/{{{c}}}_{{\rm{o}}}$, and in air ${{\mathcal{M}}}_{{\rm{Air}}}^{u}={L}_{{\rm{Air}}}^{u}\left(f\right)f/{{\rm{c}}}_{{\rm{o}}}$, respectively, and note that both expressions are now completely independent of frequency. We use these quantities as a ‘cavity configuration’ space, defining the number of optical cycles in air and quartz to analyze the resultant cavity resonances (see Fig. 4d and Figs. S15 and 16) by using the formalism described in the following.

We consider next the coupling of these two families of resonances in our hybrid cavities. We solve, as a first step, for the eigenmodes of a sub-cavity consisting of only the air gap and a single electro-optic crystal, using a coupling block matrix of the form (see Figs. S14a, 15):

M.23 $${{\mathbf{A}}}_{{\rm{sub}}}={{\boldsymbol{\mathcal{M}}}}_{{\rm{EO}}}^{T}\oplus {{\boldsymbol{\mathcal{M}}}}_{{\rm{Air}}}^{U}+C{\bf{{K}}}$$

This matrix is written in the direct-sum space of cavity configuration subspaces. That is, we consider the independent variables of ${L}_{{\rm{Air}}}$ and ${L}_{{\rm{EO}}}$, and use the coupling matrix $C{\bf{K}}$ to identify the coupled-cavity conditions (i.e. eigenvalues ${L}_{{\rm{Air}}}$ and ${L}_{{\rm{EO}}}$) that satisfy some particular cavity frequency. The diagonal sub-blocks are constructed using the bare resonances in the subspaces $T$ and ${U}$:

M.24 $${{\mathcal{M}}}_{{\rm{EO}};i,{{i}}}^{T}{{=}}\frac{2T\left({{i}}\right)-1}{4},\quad{{\mathcal{M}}}_{{\rm{Air}};i,{{i}}}^{{U}}{{=}}\frac{U\left({{i}}\right)}{2}$$

The coupling between these two subspaces is achieved using a single coupling constant $C$, and a matrix ${{\bf{K}}}$ which is filled with the value one in the ‘cross space’ sub-blocks between $T$ and $U$, and is filled with zeros in the diagonal blocks corresponding to $T$ and $U$ (Fig. S14a).

The coupling matrix ${{\bf{A}}}_{{\rm{sub}}}$ is used to solve for the eigenvectors (i.e. new resonance conditions) in the coupled sub-cavity system, according to (Fig. S13b):

M.25 $${{{\bf{A}}}}_{{\rm{sub}}}{{\boldsymbol{a}}}_{{\rm{sub}}}^{l}={{{\mathcal{M}}}}^{l}_{{\rm{sub}}}{{\boldsymbol{a}}}_{{\rm{sub}}}^{l}, \quad {{\bf{V}}}_{{\rm{sub}}}^{-1}{{{\bf{A}}}}_{{\rm{sub}}}{{{\bf{V}}}}_{{\rm{sub}}}={{\boldsymbol{\mathcal{M}}}}_{{\rm{sub}}}^{T\oplus U}$$

where ${{\bf{V}}}_{{\rm{sub}}}$ is the matrix of eigenvectors corresponding to ${{\bf{A}}}_{{\rm{sub}}}$, which executes the change of basis from the uncoupled resonances to the coupled ones.

To obtain the eigenvectors for the entire hybrid cavity, we subsequently couple the already-obtained sub-cavity eigenvectors to the basis vectors (resonances) in the remaining EO crystal, using the full cavity-configuration space ${T}_{1}\oplus U\oplus {T}_{2}$. We consider the full coupling matrix, written as follows:

M.26 $${\bf{A}}={\boldsymbol{\mathcal{M}}}_{{\rm{sub}}}^{{T}_{1}\oplus U}{{\oplus}}\,{\boldsymbol{\mathcal{M}}}_{{\rm{EO}}}^{{{T}}_{2}}+C{{{\bf{K}}}}_{{\rm{P}}}$$

where the matrix ${{{\bf{K}}}}_{{\mathrm{P}}}$ replaces the matrix ${{\bf{K}}}$ used in ${{\bf{A}}}_{{\rm{sub}}}$ and the values of one are replaced with the integrated projection of the sub-cavity eigenvectors onto the sub-space of air (Fig. S14c), which act as a weighting factor multiplied with the same coupling constant $C$ used in ${{\bf{A}}}_{{\rm{sub}}}$. The eigenvectors of this full coupling matrix correspond to the physically-allowed air gap sizes and EO crystal lengths which correspond to any given frequency (Figs. S16d–f). We also extract the characters (${\chi }_{{\rm{Air}}},{\chi }_{{\rm{EO}}}$) of the eigenvectors obtained from ${\bf{A}}$ by analyzing the projection of these eigenvectors onto the relevant subspaces (Fig. S14d, which we use to color-code the eigenvalue dispersions in Figs. S15 and 16).

We identify that it is sufficient to consider eigenvalues only in the range of ${{\mathcal{M}}}_{{\rm{EOC}}}$ and ${{\mathcal{M}}}_{{\rm{Air}}}$ between 0 and 0.5, constituting an ‘irreducible space’, thereby taking full advantage of the periodicity in the cavity-configuration space (see e.g. Fig. 4d, and Figs. S15b, d, f, and S16d, e). Thus, for every desired pair of frequency and EO crystal length, we compute the number of optical cycles in the EO crystal, ${{\mathcal{M}}}_{{\rm{EO}}}={L}_{{\rm{EO}}}f/{{{c}}}_{{\rm{o}}}$, and identify the unique corresponding eigenvector in the irreducible space. Finally, we identify the value of ${{\mathcal{M}}}_{{\rm{Air}}}$ for the identified eigenvector, as well as the higher-order modes which intersect (i.e. ${{\mathcal{M}}}_{{\rm{Air}}}$ plus half-integer multiples). In this way, we are able to identify all experimentally-observed cavity eigenfrequencies by investigating only the eigenvectors in the irreducible space. Importantly, the entire cavity dispersion is therefore dictated by a single coupling constant. However, when converted back to an experimentally-relevant basis, e.g. THz frequency vs. air gap size, the coupling constant will have an inverse-frequency dependence, as is evident in e.g. Fig. 4a, and Figs. S15g, S16f.

In summary, we have seen that all cavity eigenfrequencies can be understood in terms of a coupling between the simple building blocks which together constitute the entire cavity. Furthermore, all eigenfrequencies are derived from an entirely frequency-independent irreducible space, determined via a single coupling constant (neglecting dispersion), thus representing the most compact representation of the rich physics demonstrated in the hybrid EOCs.

### (M10) Hybrid cavity—effects of refractive index

Through a systematic study of the effect of refractive index using the field-based and coupled-oscillator models, we identify that there is a strong trade-off for using more sensitive EO crystals, such as zinc telluride (ZnTe) or gallium phosphide (GaP). We observe that the interaction strength in the coupled-oscillator model scales roughly with the transmission coefficient between air and the EO crystal (SI Fig. 17 d). Thus, for higher refractive indices, the frequency range in which air-crystal coupling occurs noticeably shrinks, leading to significantly more prominent features in cavity dispersion when tuning the air gap size (see Figs. S15g, and S16f).

Because strong coupling is ideally studied when a resonance of interest is energetically aligned with an EO crystal resonance, due to the relationship between light-matter coupling strength and the interfacial field, we identify quartz as the ideal electro-optic platform of choice for hybrid EOC studies of light-matter interaction, as the large refractive indices of ZnTe and GaP are likely to dominate the hybrid cavity electro-optical features.

## Supplementary information

Updated Supplementary Material

## Data Availability

All data used to generate the conclusions presented in this paper are made publicly available^59^. In addition, we provide our implementation of the cavity-correction function, and example codes for both the cavity-field and coupled-oscillator physical models used to describe the hybrid cavity optical responses.
