# Supplementary material for: Electro-optic cavities for in-situ measurement of cavity fields
Source: Light Sci Appl. 2025 Feb 6;14:69. doi: 10.1038/s41377-024-01685-x (PMC11799363; doi:10.1038/s41377-024-01685-x)
Supplement: Supplementary file 1 — Updated Supplementary Material [file 41377_2024_1685_MOESM1_ESM.pdf]

Supplementary Information for
Electro-Optic Cavities for In-Situ Measurement of Cavity Fields

Michael S. Spencer[1*], Joanna M. Urban[1], Maximilian Frenzel[1], Niclas S. Mueller[1], Olga Minakova[1], Martin Wolf[1], Alexander Paarmann[1], Sebastian F. Maehrlein[1,2,3*]

1. Department of Physical Chemistry, Fritz Haber Institute of the Max Planck Society, 14195 Berlin, Germany
2. Helmholtz-Zentrum Dresden-Rossendorf, Institute of Radiation Physics, 01328 Dresden, Germany
3. Technische Universität Dresden, Institute of Applied Physics, 01062 Dresden, Germany

*email: maehrlein@fhi-berlin.mpg.de, spencer@fhi-berlin.mpg.de

Table of Contents

Supplementary Discussion 1: Optical Properties of Gold Films on z-cut α-Quartz 1
Supplementary Discussion 2: Cavity Electric Fields: Travelling- and Standing-Wave Pictures 3
Supplementary Discussion 3: Cavity Electro-Optic Sampling – Extended Discussion 6
Supplementary Discussion 4: Cavity Electro-Optic Sampling – Role of Phase Mismatch in Experimental EOC Spectra 7
Supplementary Discussion 5: Comparing in-Situ Cavity Fields with Transmitted Fields 8
Supplementary Discussion 6: Probe Scattering – Field Calibration 10
Supplementary Discussion 7: Cavity Field Model 11

Figure S1: Internal and External Reflections from Cavity Mirrors 1
Figure S2: Optical Properties of Cavity Mirrors 2
Figure S3: Cavity Fields: Propagation Direction 4
Figure S4: Cavity Fields: Spatial Structure, Standing Waves 5
Figure S5: Full (Cross-Propagating) Cavity Correction Function 6
Figure S6: Phase Mismatch Function and Quartz Length 7
Figure S7: THz Transmission Studies of Quartz Cavities & Comparison w/ EOC Sampling 9
Figure S8: Gold Island Scattering 10
Figure S9: Balanced Detection of Electro-Optic Cavity Signal 12
Figure S10: Cavity Correction Function 13
Figure S11: Gold Film Mirror Optical Properties & Dispersion 14
Figure S12: Cavity-Field Model 15
Figure S13: Cavity Momentum Dispersion 16
Figure S14: Coupled-Oscillator Model – Coupling Matrix Formalism 17
Figure S15: Coupled-Oscillator Model – Partial Cavity 18
Figure S16: Coupled-Oscillator Model – Partial Cavity 19
Figure S17: Effect of Electro-Optic Crystal Refractive Index 20

Table S1: Quartz Dielectric Function 21
Table S2: Quartz 2nd Order Nonlinear Susceptibility 21

References 21

Supplementary Discussion 1

Optical Properties of Gold Films on z-cut α-Quartz

We investigate here the dispersive optical properties of the gold films deposited onto the quartz crystals using a three-layer dielectric model. We consider the reflection or transmission of a pulse from the two interfaces, i.e. the air-gold and gold-quartz interfaces (see Fig. S1 below), calculated at normal incidence. Only the principal pulse is considered, i.e. not the subsequent cavity reflections within quartz, as these are discussed subsequently in Supplementary Discussion 2. The separation of these two optical processes is justified because the gold film is deeply sub-wavelength at the thicknesses considered here.

In the following analysis, we have suppressed the variable with respect to frequency, for brevity (see Fig. S2), but all transmission and reflections are inherently a function of frequency due to the dispersion in the refractive indices of quartz and gold. We now report all possible terms that exist in the transmission and reflection regions, considering a pulse propagating starting from either outside (external r_{ext}, t_{ext}, panel a) or inside (internal: r_{int}, t_{int}, panel b) of the cavity:

$$r_{\text{ext}} \equiv \frac{E_{\text{ref}}}{E_{\text{inc}}^{\text{ext}}} = r_{\text{Air,Au}} + t_{\text{Au,Air}} r_{\text{Au,Qtz}} t_{\text{Air,Au}} e^{2i\phi} + t_{\text{Au,Air}} r_{\text{Au,Air}} r_{\text{Au,Qtz}}^2 t_{\text{Air,Au}} e^{4i\phi} + t_{\text{Au,Air}} r_{\text{Au,Air}}^2 r_{\text{Au,Qtz}}^3 t_{\text{Air,Au}} e^{6i\phi} + \cdots \quad (S1.1)$$

$$t_{\text{ext}} \equiv \frac{E_{\text{trans}}}{E_{\text{inc}}^{\text{ext}}} = t_{\text{Au,Qtz}} t_{\text{Air,Au}} e^{i\phi} + t_{\text{Au,Qtz}} r_{\text{Au,Air}} r_{\text{Au,Qtz}} t_{\text{Air,Au}} e^{3i\phi} + t_{\text{Au,Qtz}} r_{\text{Au,Air}}^2 r_{\text{Au,Qtz}}^2 t_{\text{Air,Au}} e^{5\phi} + \cdots \quad (S1.2)$$

$$r_{\text{int}} = \frac{E_{\text{ref}}}{E_{\text{inc}}^{\text{int}}} \equiv r_{\text{Qtz,Au}} + t_{\text{Au,Qtz}} r_{\text{Au,Air}} t_{\text{Qtz,Au}} e^{2i\phi} + t_{\text{Au,Qtz}} r_{\text{Au,Qtz}} r_{\text{Au,Air}}^2 t_{\text{Qtz,Au}} e^{4i\phi} + t_{\text{Au,Air}} r_{\text{Au,Air}}^2 r_{\text{Au,Qtz}}^3 t_{\text{Air,Au}} e^{6i\phi} + \cdots \quad (S1.3)$$

$$t_{\text{int}} = \frac{E_{\text{trans}}}{E_{\text{inc}}^{\text{int}}} \equiv t_{\text{Au,Air}} t_{\text{Qtz,Au}} e^{i\phi} + t_{\text{Au,Air}} r_{\text{Au,Qtz}} r_{\text{Au,Air}} t_{\text{Qtz,Au}} e^{3i\phi} + t_{\text{Au,Air}} r_{\text{Au,Qtz}}^2 r_{\text{Au,Air}}^2 t_{\text{Qtz,Au}} e^{5i\phi} + \cdots \quad (S1.4)$$

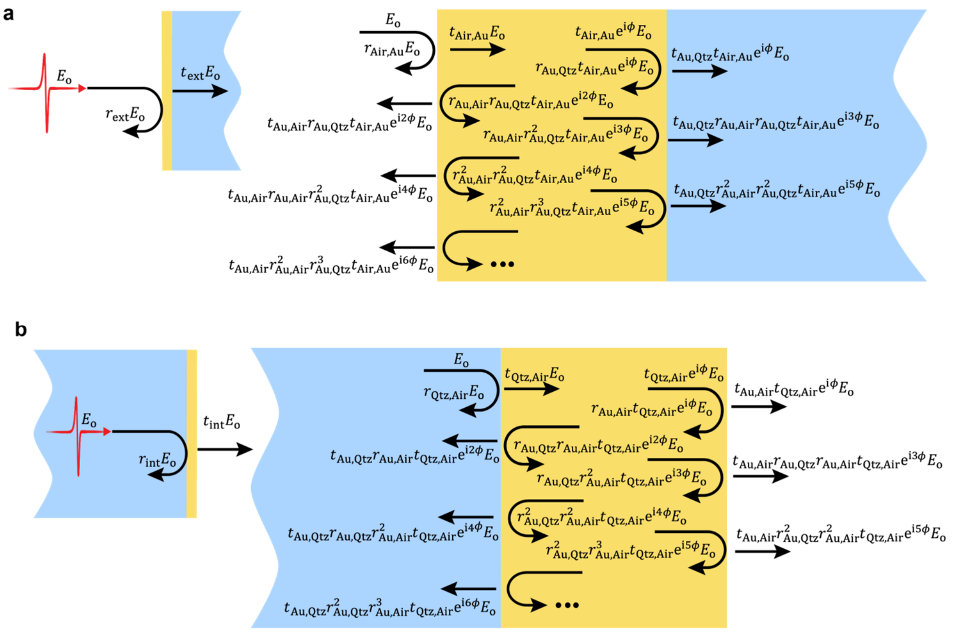

Fig. S1| Internal and External Reflections From Cavity Mirrors. a, The external reflection and transmission of a pulse (left) is diagrammed (right) as resulting from all possible internal reflections and transmissions from the thin gold layer. The summation of all terms propagating left constitutes the external reflection coefficient (S1.1), and all rightward propagating constitutes the external transmission coefficient (S1.2). **b,** The internal reflection and transmission of a pulse (left) is diagrammed (right) as resulting from all possible internal reflections and transmissions from the thin gold layer. The summation of all terms propagating left constitutes the internal reflection coefficient (S1.3), and all rightward propagating constitutes the external transmission coefficient (S1.4).

We define the (frequency-dependent) transmission or reflection coefficients in terms of the ratio of the spectrum of the transmitted or reflected pulse with the spectrum of the relevant incident pulse. The reflection and transmission coefficient at a given interface is therefore given by $r_{\text{i,j}} = (n_{\text{i}} - n_{\text{j}})/(n_{\text{i}} + n_{\text{j}})$ and $t_{\text{i,j}} = 2n_{\text{i}}/(n_{\text{i}} + n_{\text{j}})$, respectively, and the phase accumulation and attenuation over the gold film length is defined as $\phi = k(\omega)d_{\text{Au}} = \omega\big(n_{\text{Au}}(\omega) + i\kappa_{\text{Au}}(\omega)\big)d_{\text{Au}}/c_{\text{o}}$, where c_{o} is the vacuum speed of light, n and κ are the real and imaginary components of the refractive index, and where we have used the plane-wave convention $E_{\text{o}}e^{i(kz-\omega t)}$ for a rightward-traveling ($+z$) plane wave. Summation using the geometric series allows the above expressions to be evaluated as the following:

$$r_{\text{ext}} \equiv \frac{E_{\text{ref}}}{E_{\text{inc}}^{\text{ext}}} = r_{\text{Air,Au}} + \frac{t_{\text{Au,Air}} r_{\text{Au,Qtz}} t_{\text{Air,Au}} e^{2i\phi}}{1 - r_{\text{Au,Air}} r_{\text{Au,Qtz}} e^{2i\phi}} \quad (S1.5)$$

$$t_{\text{ext}} \equiv \frac{E_{\text{trans}}}{E_{\text{inc}}^{\text{ext}}} = \frac{t_{\text{Au,Qtz}} t_{\text{Air,Au}} e^{i\phi}}{1 - r_{\text{Au,Air}} r_{\text{Au,Qtz}} e^{2i\phi}} \quad (S1.6)$$

$$r_{\text{int}} = \frac{E_{\text{ref}}}{E_{\text{inc}}^{\text{int}}} = r_{\text{Qtz,Au}} + \frac{t_{\text{Au,Qtz}} r_{\text{Au,Air}} t_{\text{Qtz,Au}} e^{2i\phi}}{1 - r_{\text{Au,Qtz}} r_{\text{Au,Air}} e^{2i\phi}} \quad (S1.7)$$

$$t_{\text{int}} = \frac{E_{\text{trans}}}{E_{\text{inc}}^{\text{int}}} = \frac{t_{\text{Au,Air}} t_{\text{Qtz,Au}} e^{i\phi}}{1 - r_{\text{Au,Qtz}} r_{\text{Au,Air}} e^{2i\phi}} \quad (S1.8)$$

These functions are plotted in Fig. S2, for various gold thickness, spanning the extremes of 0 to 200 nm, using the complex, frequency-dependent refractive indices computed from the quartz dielectric function used in Table S1 and the Drude Parameters for gold reported in the Methods M3. We display the absolute values of the coefficients in Fig. S2, but note that they are generally complex. We also note that there is significant dispersion in these transmission functions (both internal and external), which is a fundamental optical feature of the multi-layer structure, and could only be eliminated in the case of a true, free-standing gold film. Therefore, pulse dispersion should be considered in any case where the gold film is deposited on a dielectric substrate, and where detailed knowledge of the precise electric field (e.g. when considering field-driven nonlinear effects) is of critical importance. In Figure S11c,d, we demonstrate the consequences of this, showing the relative increase in pulse FWHM and central frequency for pulses transmitted into and out of the cavity, presenting a technical hurdle to connecting an externally-measured field with the internal one within a cavity. This effect persists until much thicker films, (d_{Au} ~ 100 nm) at which point the optical path length, $\lambda = n(\Omega_{\mathrm{THz}})\lambda_o$, in the gold film becomes comparable to the free-space wavelength in the few-THz region. In this limit, the absorption in gold becomes the dominant feature in the spectral properties of the multi-layer interface.

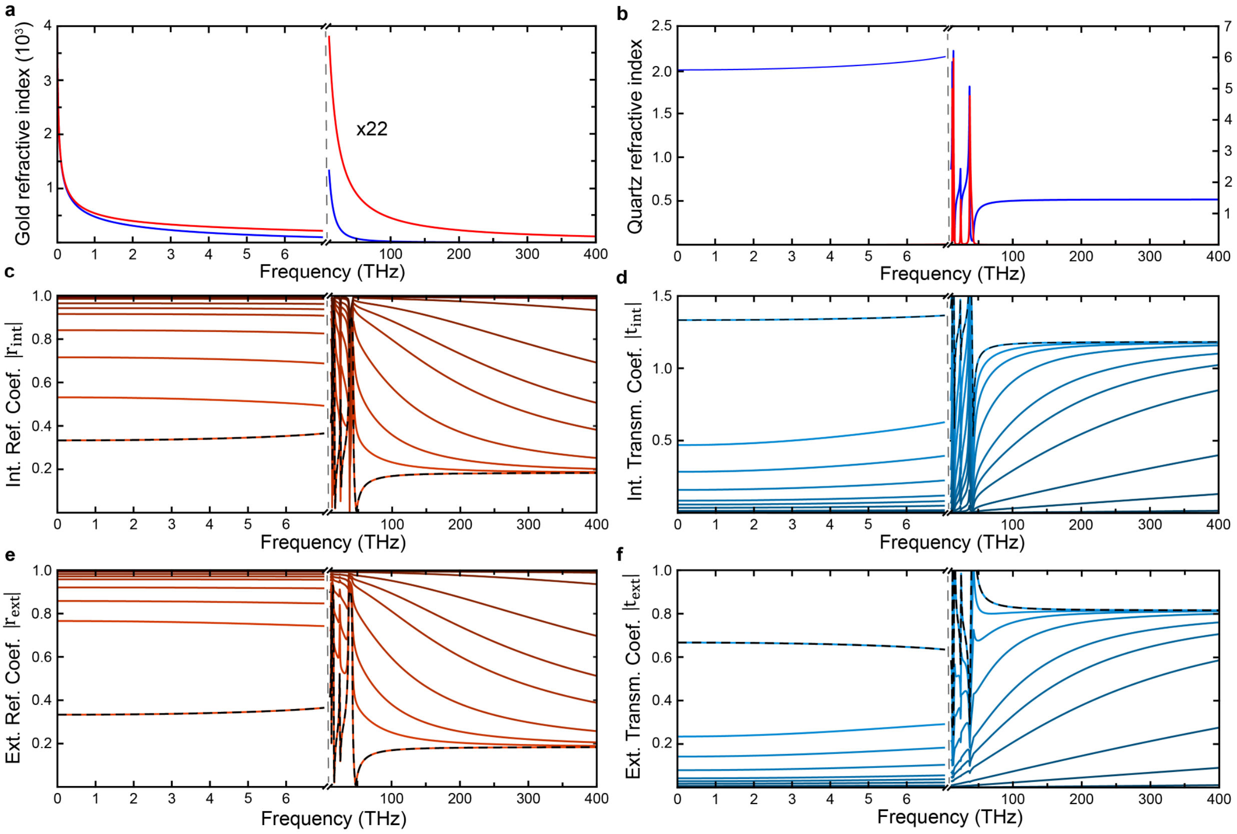

Fig. S2| Optical Properties of Cavity Mirrors. **a**, Complex dielectric function of gold. The real part is displayed in blue, and the imaginary part in red. **b**, Complex refractive index function of quartz. The real part is displayed in blue, and the imaginary part in red. **c**, Absolute value of the internal field reflection coefficient (S1.7; absolute value) as a function of gold film thickness, plotted for the values 0, 0.5, 1, 2, 4, 6, 10, 25, 50, 100, and 200 nm, from bottom to top. The result for $n_{Qtz,Air}$ is plotted as well (dotted black line), in correspondence with the d_{Au} = 0 nm result. **d**, Absolute value of the internal field transmission coefficient (S1.8; absolute value) is plotted for the same thickness parameters as in panel c. **e**, Absolute value of the external field reflection coefficient (S1.5; absolute value) is plotted for the same thickness parameters as in panel c. **f**, Absolute value of the external field transmission coefficient (S1.6; absolute value) is plotted for the same thickness parameters as in panel c.

Supplementary Discussion 2

Cavity Electric Fields: Travelling- and Standing-Wave Pictures

One way to describe an electric field inside of a cavity is the picture of a traveling pulse, experiencing successive reflections at every interface. The complementary picture to the traveling pulse is that of the 'cavity modes', obtained via Fourier transformation, which can function as an eigenbasis of the cavity electric field. The equivalence of these pictures is demonstrated in this section. We write the incident THz pulse, $E_{\mathrm{THz}}^{\mathrm{inc}}(t,z)$, - here, in terms of its Fourier-transform $E_{\mathrm{THz}}^{\mathrm{inc}}(\omega,k_z)$ - as a simplified, one-dimensional function of time (frequency) and space (momentum), where the inclusion of the momentum, $k_z(\omega)=\omega n(\omega)/c_o$, is necessary to include effects of dispersion on the pulse inside the cavity medium:

$$E_{\mathrm{THz}}^{\mathrm{inc}}(t,z)=\frac{1}{\sqrt{2\pi}}\int E_{\mathrm{THz}}^{\mathrm{inc}}(\omega,k_z)\,\mathrm{e}^{\mathrm{i}(k_z(\omega)z-\omega t)}\,d\omega \tag{S2.1}$$

Figure S3 demonstrates how a wave traveling within the cavity will progressively decrease in amplitude, due to the partial transmission of the field upon reflection at the imperfect gold mirrors. Each partially reflected or transmitted wave amplitude is related to the original incident wave, using both the internal and external reflection and transmission field coefficients detailed in Supplementary Discussion 1.

To compare with electro-optic sampling measurements, the cavity field as a function of both space and frequency must be determined. To achieve this, we infer the electric field at an arbitrary cavity position, and account for the cavity end-mirror reflections, as a function of time. This is achieved, while including the effects of dispersion, using the Fourier transform definition (Equation S2.1):

$$E_{\mathrm{THz}}^{\mathrm{cav}}(t,z)=t_{\mathrm{ext}}\left[\sum_{q=0}^{\infty}{r_{\mathrm{int}}}^{2q}E_{\mathrm{THz}}^{\mathrm{inc}}\left(t-q\tau_{\mathrm{RT}}(\omega)+\frac{zn(\omega)}{\mathrm{c_o}},z\right)+\sum_{q=0}^{\infty}{r_{\mathrm{int}}}^{2q+1}E_{\mathrm{THz}}^{\mathrm{inc}}\left(t-\frac{(2q+1)\tau_{\mathrm{RT}}(\omega)}{2}-\frac{zn(\omega)}{\mathrm{c_o}},z\right)\right] \tag{S2.2}$$

where the first summation is all forward-propagating instances of the pulse passing at some position z in the cavity, and the second summation is the backward-propagating instances at that same position, and where $\tau_{\mathrm{RT}}(\omega)=2L_{\mathrm{cav}}\mathrm{n}(\omega)/\mathrm{c_o}$ is the cavity round-trip time. By Fourier transforming this expression, we identify the spatially-resolved cavity spectrum as it relates to the incident pulse spectrum:

$$E_{\mathrm{THz}}^{\mathrm{inc}}(\omega,z)=E_{\mathrm{THz}}^{\mathrm{inc}}(\omega)t_{\mathrm{ext}}\left[\sum_{q=0}^{\infty}r_{\mathrm{int}}^{2q}\,\mathrm{e}^{\mathrm{i}(k_z(\omega)z-q\omega\tau_{\mathrm{RT}})}+\sum_{q=0}^{\infty}r_{\mathrm{int}}^{2q+1}\,\mathrm{e}^{\mathrm{i}\left(-k_z(\omega)z-\left(\frac{2q+1}{2}\right)\omega\tau_{\mathrm{RT}}\right)}\right] \tag{S2.3}$$

The infinite summations can be carried out analytically using the geometric series definition(s), yielding:

$$\frac{E_{\mathrm{THz}}^{\mathrm{cav}}(\omega,z)}{E_{\mathrm{THz}}^{\mathrm{inc}}(\omega,z)}=t_{\mathrm{ext}}\left[\frac{\mathrm{e}^{\mathrm{i}k_z(\omega)z}}{1-r_{\mathrm{int}}^2\mathrm{e}^{-\mathrm{i}\omega\tau_{\mathrm{RT}}(\omega)}}+\frac{r_{\mathrm{int}}\mathrm{e}^{-\mathrm{i}\left(k_z(\omega)z+\frac{\omega\tau_{\mathrm{RT}}(\omega)}{2}\right)}}{1-r_{\mathrm{int}}^2\mathrm{e}^{-\mathrm{i}\omega\tau_{\mathrm{RT}}(\omega)}}\right]=\frac{t_{\mathrm{ext}}\left(\mathrm{e}^{\mathrm{i}k_z(\omega)z}+r_{\mathrm{int}}\mathrm{e}^{-\mathrm{i}k_z(\omega)z}\mathrm{e}^{\frac{-\mathrm{i}\omega\tau_{\mathrm{RT}}(\omega)}{2}}\right)}{1-r_{\mathrm{int}}^2\mathrm{e}^{-\mathrm{i}\omega\tau_{\mathrm{RT}}(\omega)}} \tag{S2.4}$$

Figure S3b displays the time-domain fields, and corresponding amplitude- and phase-resolved spectrum of the fields evaluated at the cavity center ($z=0$), comparing the total field to the purely forward- or backwards-propagating pulse summations, for the choices of d_{Au} = 2 nm, and d_{Au} = 10, all evaluated for a round-trip time τ_{RT} ≈ 0.6 ps, corresponding to the L_{Qtz} = 44 μm cavity. Note that for the simple derivation outlined above, we have suppressed any frequency content in the reflection and transmission coefficients for simplicity, but however use the full, dispersive functions outlined in Supplementary Discussion 1 when evaluating equation S2.4 to depict the fields in Fig. S3. It is evident that constructive interference among the single-pulse Fourier transforms occurs only at the cavity resonance conditions, i.e. the frequencies which are integer multiples of the inverse of the round-trip time. In addition, we can see that only half of the modes are visible at the cavity position $z=0$, due to perfect cancellation of Fourier transforms in the forward- and backward-propagating direction, as detailed further later.

We now seek to show that this cavity response derived above can also be understood equivalently as a sum of cavity eigenmodes. To start, we show the cavity intensity response, considering either the total cavity field, or the forward/reverse-propagating cavity intensities:

$$\left|\frac{E_{\mathrm{THz}}^{\mathrm{cav}}(\omega,z)}{E_{\mathrm{THz}}^{\mathrm{inc}}(\omega,z)}\right|^2=\frac{|t_{\mathrm{ext}}|^2\left(1+2|r_{\mathrm{int}}|\cos\left(k(\omega)z+\frac{\omega\tau_{\mathrm{RT}}(\omega)}{2}\right)+|r_{\mathrm{int}}|^2\right)}{1-2|r_{\mathrm{int}}|^2\cos(\omega\tau_{\mathrm{RT}})+|r_{\mathrm{int}}|^4} \tag{S2.5}$$

$$\left|\frac{E_{\mathrm{THz}}^{\mathrm{cav,f}}(\omega,z)}{E_{\mathrm{THz}}^{\mathrm{inc}}(\omega,z)}\right|^2=\frac{|t_{\mathrm{ext}}|^2}{1-2|r_{\mathrm{int}}|^2\cos(\omega\tau_{\mathrm{RT}}(\omega))+|r_{\mathrm{int}}|^4},\quad\left|\frac{E_{\mathrm{THz}}^{\mathrm{cav,b}}(\omega,z)}{E_{\mathrm{THz}}^{\mathrm{inc}}(\omega,z)}\right|^2=\frac{|t_{\mathrm{ext}}|^2|r_{\mathrm{int}}|^2}{1-2|r_{\mathrm{int}}|^2\cos(\omega\tau_{\mathrm{RT}}(\omega))+|r_{\mathrm{int}}|^4} \tag{S2.6}$$

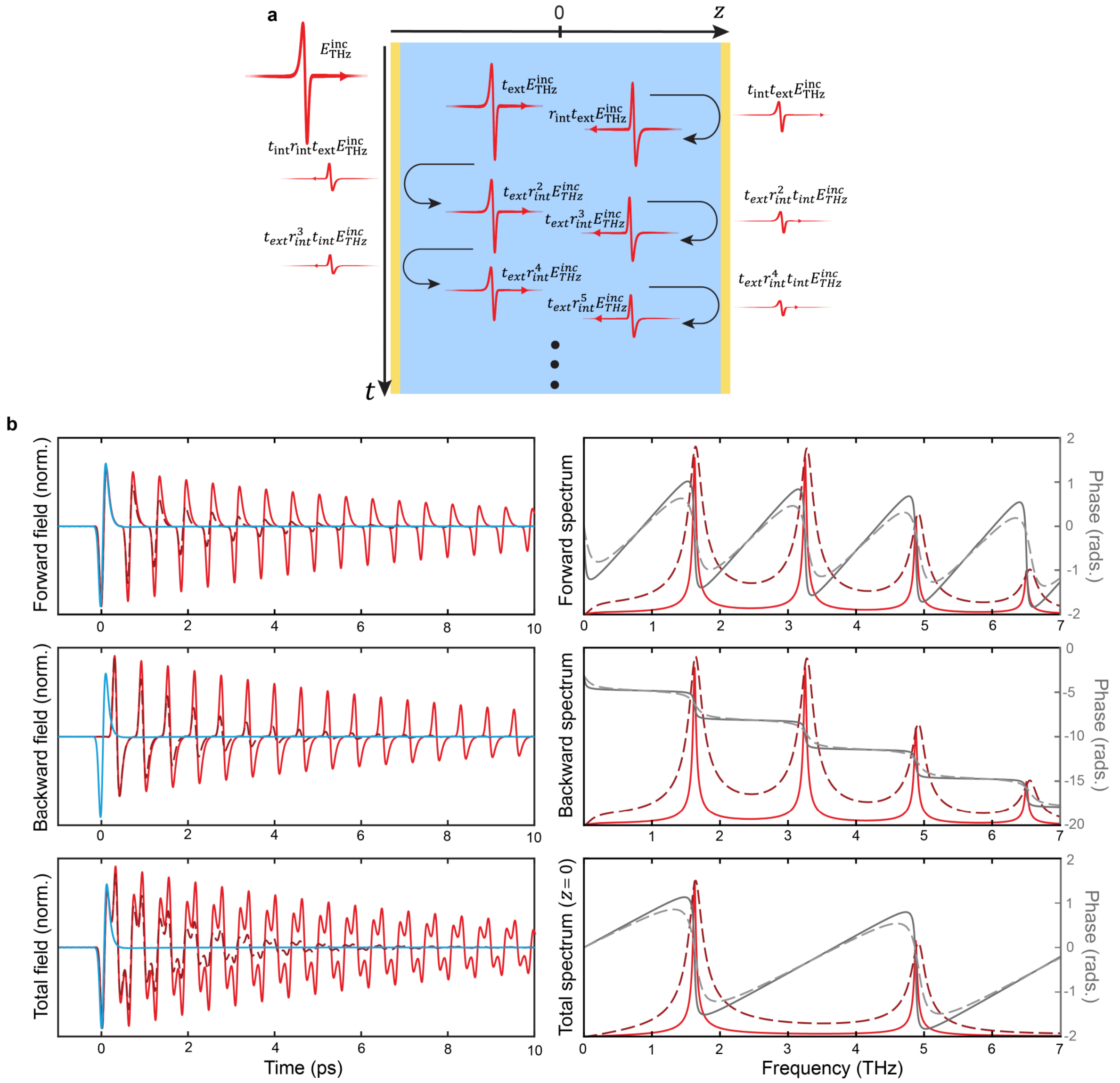

Fig. S3| Cavity Fields - Theory. a, The internal cavity field is defined by the principal THz pulse reflecting internally within the cavity, partially transmitting at every encounter with the end mirrors. **b**, We plot the normalized, time-domain cavity electric fields (left), distinguishing between the forward-propagating instances (top) of the principal pulse (blue) , the backwards-propagating instances (center), and their sum (bottom), in every case for gold film thicknesses of 2 (dashed lines) and 10 nm (solid lines). The corresponding spectra are shown on the right, represented in terms of the absolute value (red), and the phase (gray).

Moving forward, we consider only the forward-propagating field, for the sake of simplicity. Using the definition of the round-trip time, we re-cast the cavity intensity response as the following:

$$\left|\frac{E_{\mathrm{THz}}^{\mathrm{cav,f}}(\omega,z)}{E_{\mathrm{THz}}^{\mathrm{inc}}(\omega,z)}\right|^2 = \frac{|t_{\mathrm{ext}}|^2}{(1-|r_{\mathrm{int}}|^2)^2 + 4|r_{\mathrm{int}}|^2 \sin^2\left(\frac{\omega L_{\mathrm{cav}}}{c}\right)} \tag{S2.7}$$

where $c = c_o/n(\omega)$ is the speed of light inside the EO crystal.

Finally, we demonstrate that the sum of all cavity modes yields an identical expression to the one shown above, following the analysis of Ismail *et al.*[1]. To start, we note that all modes share the same loss rate as that of the travelling pulse, which is defined by the round-trip field loss rate and the cavity round trip time, as follows:

$$|r_{\mathrm{int}}|^2 = \mathrm{e}^{-\frac{\tau_{\mathrm{RT}}}{\tau_{\mathrm{loss}}}} \;\rightarrow\; \frac{1}{\tau_{\mathrm{loss}}} = -\frac{\log(|r_{\mathrm{int}}|^2)}{\tau_{\mathrm{RT}}} \tag{S2.8}$$

The cavity modes have frequencies which correspond to integer multiples of the inverse of the cavity round trip time, when neglecting dispersion, i.e. $f_{\mathrm{cav}}^{q} = \pm\frac{q\,\mathrm{c_o}}{n(f_{cav}^{q})L_{\mathrm{Cav}}}$, where q is the mode index, which spans all integers. Using this loss rate relation, the electric field of these modes is re-written as:

$$E_{\pm q}(t) = E_{\pm q}(0)\, \mathrm{e}^{\mp 2\pi \mathrm{i} f_{\mathrm{cav}}^{q} t}\, \mathrm{e}^{-\frac{t}{\tau_{\mathrm{loss}}}} \tag{S2.9}$$

From this, we compute the Fourier transform of the modes, which has the typical complex field Lorentzian form:

$$E_{\pm q}(\omega) = \frac{E_{\pm q}(0)}{\sqrt{2\pi}} \int_0^\infty \mathrm{e}^{-\mathrm{i}\omega_{\mathrm{cav}}^{q} t} \mathrm{e}^{-\mathrm{t}/\tau_{\mathrm{loss}}} \mathrm{e}^{\mathrm{i}\omega t} = \frac{E_{\pm q}(0)}{\sqrt{2\pi}} \left(\frac{1}{\mathrm{i}(\omega \mp \omega_q) - \frac{1}{\tau_{\mathrm{loss}}}} \right) \tag{S2.10}$$

From this, the cavity spectral intensity for a particular mode is identified:

$$\left|\frac{E_{\pm q}(\omega)}{E_{\pm q}(0)}\right|^2 = \frac{1}{2\pi}\left(\frac{1}{\left(\omega \mp \omega_q\right)^2 - \frac{1}{\tau_{\text{loss}}^2}}\right) \tag{S2.11}$$

Normalizing against the linewidth, and introducing the term $\delta_{\text{out}} = -\log(r_{\text{int}}^2) = \tau_{\text{RT}}/\tau_{\text{loss}}$, representing the fractional field reduction per round trip, we write the normalized cavity mode response as follows:

$$R_{\pm q} \equiv \frac{1}{\tau_{loss}}\left|\frac{E_{\pm q}(\omega)}{E_{\pm q}(0)}\right|^2 = \frac{1}{2\pi}\frac{\tau_{\text{RT}}}{\delta_{\text{out}}}\frac{\left(\frac{\delta_{\text{out}}}{\tau_{RT}}\right)^2}{\left(\omega \mp \omega_q\right)^2 + \left(\frac{\delta_{out}}{\tau_{RT}}\right)^2} \tag{S2.12}$$

After several manipulations of the above expression[1], it can be shown that:

$$\left|\frac{E_{\text{THz}}^{\text{cav,f}}(\omega, z)}{E_{\text{THz}}^{\text{inc}}(\omega, z)}\right|^2 = \sum_{q=-\infty}^{\infty}\frac{1}{\tau_{\text{RT}}}R_{\pm q} = \frac{|t_{\text{ext}}|^4}{4r_{\text{int}}^2 \sin^2\left(\frac{\omega n(\omega) L_{\text{cav}}}{c_0}\right) + (1 - r_{\text{int}}^2)^2} \tag{S2.13}$$

Note that this expression is identical to the one derived using the time-domain analysis of a travelling pulse. Therefore, it has been demonstrated that the summation of all optical modes within the cavity produces the Fabry-Perot response derived from the travelling pulse formalism, showing the complete equivalence of the two perspectives.

Finally, we observe that the summation of the forwards- and backwards-propagating waves' interference leads to the familiar standing modes in space. This is demonstrated by evaluating the frequency-domain cavity field (Equation S2.4) at the resonance condition(s). The momenta of the cavity modes are given by $k_z(\omega_q) = \pi q/L_{\text{cav}}$, with corresponding wavelengths of $\lambda_q = 2nL_{\text{cav}}/q$, where we approximate without dispersion, leading to cavity mode frequencies $\omega_q = q\frac{\pi c_0}{L_{\text{cav}} n}$. Using these expressions, the cavity field simplifies to the following:

$$\frac{E_{\text{THz}}^{\text{cav}}(\omega, z)}{E_{\text{THz}}^{\text{inc}}(\omega)} = t_{\text{ext}}\frac{e^{i\left(\frac{\pi q}{L_{\text{cav}}}\right)z} + r_{\text{int}}e^{-i\left(\frac{\pi q}{L_{\text{cav}}}\right)z}e^{-i\pi q}}{1 - r_{\text{int}}^2} \tag{S2.14}$$

We thus arrive at two different sets of solutions, assuming high internal reflectivites, depending on whether q is an even or odd integer:

$$\text{E}_{\text{THz}}^{\text{cav}}(\omega^{\text{even}}, z) \propto \cos\left(\frac{\pi q}{L_{\text{cav}}}z\right), \qquad \text{E}_{\text{THz}}^{\text{cav}}(\omega^{\text{odd}}, z) \propto \text{i}\sin\left(\frac{\pi q}{L_{\text{cav}}}z\right) \tag{S2.15}$$

These standing waves, and the Lorentzian decomposition of the Fabry-Perot response are both demonstrated in Fig. S4. We note also the interesting property that in the case of no cavity, i.e. just an electro-optic crystal, that there is no loss in spectral amplitude for the cavity field at the constructive-interference conditional frequencies, if sampled at the correct cavity positions (see e.g. Fig. S4b). Furthermore, we observe that once the cavity mirrors are included, the drop in peak spectral amplitude corresponds to precisely $1/n_{\text{EO}}$.

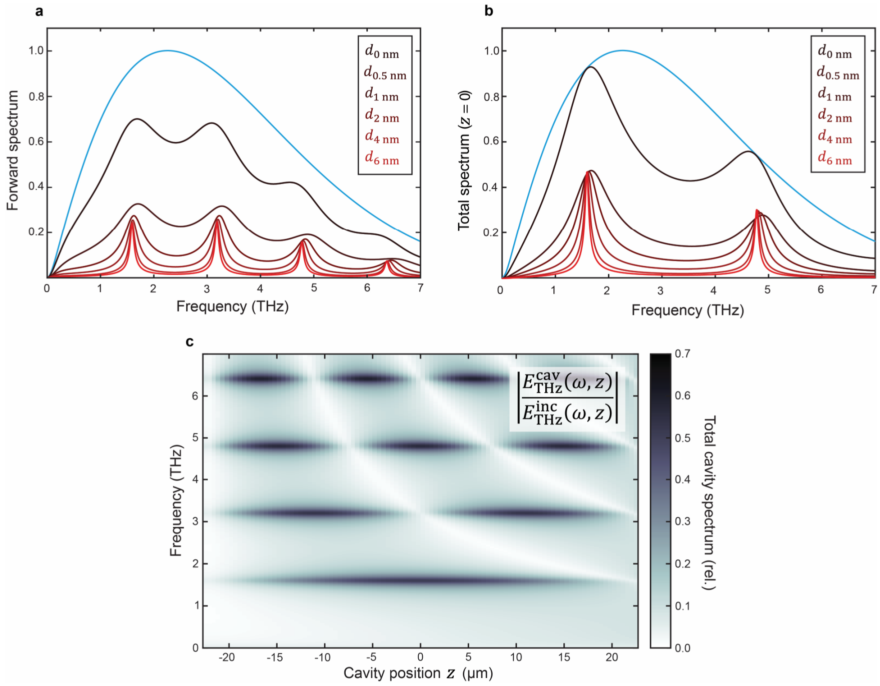

Fig. S4| Cavity Fields: Spatial Structure, Standing Wave. **a**, The calculated cavity spectra for strictly forward-propagating principal pulses are shown for various gold film thickness, all compared and normalized against the incident pulse spectrum (blue). **b**, The calculated cavity spectra for the total cavity field (i.e. forward- and backwards-propagating; Eqtn. $S2.14$) is displayed, at the same gold thicknesses considered in panel a, computed at the cavity center ($z = 0$) **c,** The total cavity spectrum is shown as a function of cavity position and THz frequency. This simulation uses a gold film thickness of 2 nm, and a quartz crystal length of 44 μm.

Supplementary Discussion 3

Cavity Electro-Optic Sampling – Extended Discussion

The cavity correction function utilized in the main text expands upon the conventional detector response function[2,3], which follows from considering a generic nonlinear wave mixing between terahertz and visible-frequency pulses, incorporating nonlinear boundary conditions and absorptive losses.[4,5] The general form of the cavity correction function is therefore understood as an extension of a conventional detector response function, except where we use the cavity electric fields of the THz and visible probing pulses to compute the nonlinear SFG/DFG polarizations in the cavity:

$$E_{\mathrm{cav}}(\omega,z) = E_{\mathrm{inc}}(\omega,z)\frac{t_{\mathrm{ext}}(\omega)\left(e^{ik(\omega)z} + r_{\mathrm{int}}(\omega)e^{-ik_z(\omega)z}\,e^{-\frac{i\omega\tau_{\mathrm{RT}}(\omega)}{2}}\right)}{1 - r_{\mathrm{int}}^2(\omega)e^{-i\omega\tau_{RT}(\omega)}} \tag{S3.1}$$

where here ω can generally refer to either the THz frequency Ω_{THz} or the visible frequency ω_{vis}, and where have distinguished between the external and internal reflection and transmission coefficients at a given frequency, due to the asymmetry at the air-gold-quartz interfaces (see Supplementary Discussion 1, & Fig. S10). The refractive index for quartz used in this work is tabulated for reference in Table S1.

The nonlinear polarization inside of the cavity is then computed in terms of the product of the THz and visible probing cavity fields[33,51] (i.e. using Eqtn. $S3.1$):

$$\frac{\partial}{\partial z}E_{\mathrm{cav}}(\omega_\pm,z) = \mathrm{i}\frac{\omega_\pm^2}{2\mathrm{c}_0^2 k(\omega_\pm)}\chi_{\mathrm{eff}}^{(2)}(\Omega_{\mathrm{THz}})\frac{t_{\mathrm{ext}}(\Omega_{\mathrm{THz}})\,t_{\mathrm{ext}}(\omega_{\mathrm{vis}})E_{\mathrm{inc}}^{\mathrm{THz}}(\omega,z)E_{\mathrm{inc}}^{\mathrm{vis}}(\omega,z)\left(\mathrm{e}^{\mathrm{i}\Delta k_{\mathrm{co}}z} + r_{\mathrm{int}}(\Omega_{\mathrm{THz}})\mathrm{e}^{-\frac{i\Omega_{\mathrm{THz}}\tau_{\mathrm{RT}}(\Omega_{\mathrm{THz}})}{2}}\mathrm{e}^{\mathrm{i}\Delta k_{\mathrm{xr}}z}\right)}{\left(1 - r_{\mathrm{int}}^2(\Omega_{\mathrm{THz}})\mathrm{e}^{-\mathrm{i}\Omega_{\mathrm{THz}}\tau_{\mathrm{RT}}(\Omega_{\mathrm{THz}})}\right)\left(1 - r_{\mathrm{int}}^2(\omega_{\mathrm{vis}})\mathrm{e}^{\mathrm{i}\omega_{\mathrm{vis}}\tau_{\mathrm{RT}}(\omega_{\mathrm{vis}})}\right)} \tag{S3.2}$$

where $\omega_\pm = \omega_{\mathrm{vis}} \pm \Omega_{\mathrm{THz}}$ is the angular frequency of the emitted (SFG/DFG) field. Note that the phase in the Fabry-Pérot transfer function for the probe field is conjugated compared to the THz transfer function – a consequence of the relative time delay definition between the THz and probe pulses. We have identified the co- and counter-propagating momentum mismatch as:

$$\begin{aligned}\Delta k_{\mathrm{co}} &= \Delta k_{\mathrm{co}}(\omega_\pm;\Omega_{\mathrm{THz}},\omega_{\mathrm{vis}}) = k(\omega_\pm) - k(\omega_{\mathrm{vis}}) \mp k(\Omega_{\mathrm{THz}}) \approx \pm(n_{\mathrm{THz}} - n_{\mathrm{vis}})\Omega_{\mathrm{THz}} \\ \Delta k_{\mathrm{xr}} &= \Delta k_{\mathrm{xr}}(\omega_\pm;\Omega_{\mathrm{THz}},\omega_{\mathrm{vis}}) = k(\omega_\pm) + k(\omega_{\mathrm{vis}}) \mp k(\Omega_{\mathrm{THz}}) \approx \pm(n_{\mathrm{THz}} + n_{\mathrm{vis}})\Omega_{\mathrm{THz}}\end{aligned} \tag{S3.3}$$

We have assumed once again that there is negligible dispersion of the refractive index at visible frequencies to arrive at the final expressions on the right-hand side of Equation $S3.3$.

In principle, we have four terms in total to consider, given the possible combinations of THz and probing pulse propagation directions. We have assumed already in writing Equation S3.1 that the reflection of the probe inside the cavity is relatively minor, eliminating the two terms arising from the backwards-travelling probing pulse. This is a relatively crude approximation, but bolstered by the fact that the probe must internally reflect twice to be finally measured in the transmission region, producing at that point a relatively very weak signal. A related approximation we make is that the SFG/DFG signal does not have the cavity reflectivity imposed upon it. Nevertheless, we observe that the principal single-cycle pulse extracted without including cross-propagation (Fig. S9d) or including it (Fig. S5 below) suggest that this level of treatment is sufficient for the moderate cavity quality factors investigated here.

The functional form of the cavity correction function displayed in the Methods is derived from a simplified form of Equation S3.1, where we do not consider the backwards-travelling THz pulse when considering the nonlinear polarization inside of the cavity. We present here a more comprehensive correction function:

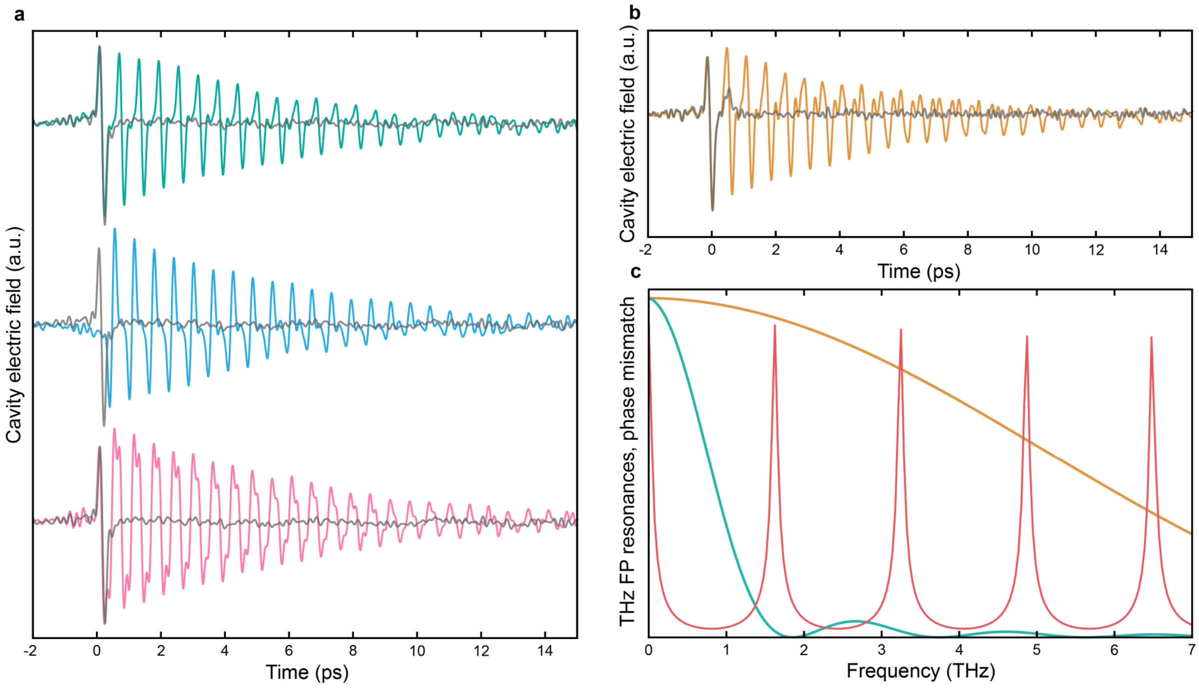

Fig. S5| Full (Cross-Propagating) Cavity Correction Function. **a**, The forward-propagating field (green, top), backwards-propagating field (blue, middle), and the sum (pink, bottom), evaluated at the cavity center (z=0), using the principal pulse (gray) obtained from the full cavity correction function. **b**, The principal pulse (gray) and cavity field (orange) obtained using the approximate form of the cavity correction function, where weak detection of the counter-propagating THz pulse is still present. **c**, The Fabry-Pérot resonances (red; L_{QTz} = 44 μm) are generally near the zeros of the counter-propagating phase mismatch function (green), plotted here in comparison with the co-propagating phase mismatch function (orange).

$$h_{fun}(\Omega_{THz}) = \chi_{eff}^{(2)}(\Omega_{THz})t_F(\Omega_{THz})\int_{\omega_{vis}} \frac{\omega_{vis}^2}{c_0^2 k(\omega_{vis})} T_{pr}(\omega_{vis},\Omega_{THz})E_{pr}^*(\omega_{vis})\,E_{pr}(\omega_{vis} \mp \Omega_{THz}) \cdots$$
$$\left(G_{co}(\omega_{vis},\Omega_{THz}) + r_{int}(\Omega_{THz})e^{-\frac{i\Omega_{THz}\tau_{RT}(\Omega_{THz})}{2}} G_{xr}(\omega_{vis},\Omega_{THz})\right) d\omega_{vis} \tag{S3.4}$$

where the new total phase mismatch term is distributed into the conventional one, $G_{co}(\omega_{vis},\Omega_{THz})$ as defined in the Methods, as well as a new one corresponding to counter-propagating THz and probing pulses:

$$G_{xr}(\omega_{vis},\Omega_{THz};L_{Qtz}) = \left(\frac{e^{i\Delta k_{xr}(\Omega_{THz},\omega_{vis})L_{Qtz}} - 1}{i\Delta k_{xr}(\Omega_{THz},\omega_{vis})}\right) \tag{S3.5}$$

We show the convolution of this field with the backwards-propagating cavity field transfer function (evaluated at z = 0). We note that this is an expected field, rather than a direct measurement, as we only very weakly measure the backwards-propagating field, as expected from the severe phase mismatch (Fig. S5c), and evidenced by the subtle differences between the extracted principal pulses with and without counter-propagating fields (Figs. S5a,b, respectively). We note also that an EO measurement will include integration over all cavity positions, in which case the phase difference between forwards- and backwards-travelling pulses is spatially dependent (see S2.2). In summary, we display the total field for purposes of demonstrating the expected cavity fields at the center of the cavity, despite electro-optic sampling primarily measuring co-propagating pulses.

Supplementary Discussion 4

Cavity Electro-Optic Sampling – Role of Phase Mismatch in Experimental EOC Spectra

We note that the effects of phase mismatch as it is defined in Equation 3 of the Methods are responsible for noise injected (i.e. division by zero) into the cavity spectrum due to the application of the cavity correction function. We depict below in Fig. S6b the normalized phase mismatch function as a function of quartz crystal thickness, where we identify the first zero-crossing in red and show this on top of the reproduced experimental cavity EO spectra from **Fig. 2b**. The normalized phase mismatch function is defined as:

$$G_{norm}(\omega_{vis},\Omega_{THz};L_{Qtz}) = \frac{e^{i\Delta k_{co}(\Omega_{THz},\omega_{vis})L_{Qtz}} - 1}{i\Delta k_{co}(\Omega_{THz},\omega_{vis})L_{Qtz}} = \frac{G(\omega_{vis},\Omega_{THz};L_{Qtz})}{L_{Qtz}} \tag{S4.1}$$

where we have used the definition of the co-propagating momentum mismatch as written in Equation S3.3.

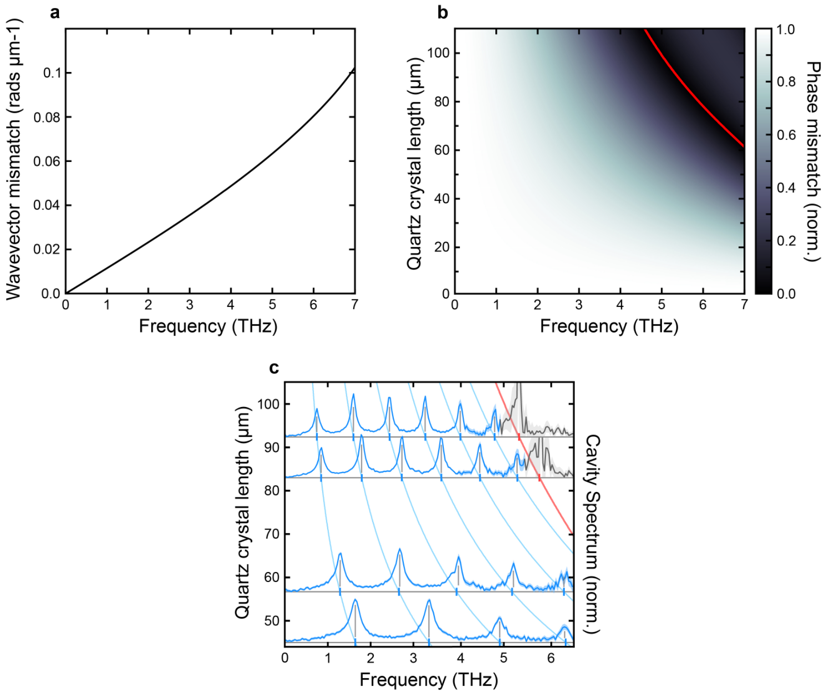

Fig. S6| Effect of Phase Mismatch Function. a, The phase mismatch is displayed as a function of THz refractive index. **b,** The (normalized) momentum mismatch function is displayed as a function of both THz frequency and quartz crystal length L_{Qtz}. The first zero of this phase mismatch function is denoted here as a red line. **c,** The EOC spectrum as a function of quartz length is reproduced from **Fig. 2b**, where we additionally overlay the first zero of the phase mismatch function, to highlight the origin of the injected noise which we exempt from our time-domain fields by application of a masking function.

Supplementary Discussion 5

Comparing in-Situ Cavity Fields with Transmitted Fields

We compare here our method of EOC sampling with conventional EO sampling of transmitted cavity fields. To identify the transmitted field, we perform electro-optic sampling with a double-focus EO-sampling geometry (see Fig. S7a), where we measure a signal without the cavity $S_{\text{ref}}(t)$ (i.e. reference scan). Afterwards, we conduct a separate measurement with the cavity in the first THz focus, obtaining $S_{\text{cav}}(t)$ (Fig. S7c). The ratio of the corresponding spectra yields the transmittance (Fig. S7d), as both the detector-response function, $h(\omega)$, and the incident field spectrum will cancel out, leaving only the transmission function $T_{\text{THz}}(\omega)$ for the quartz cavity (Eq. S5.2; data visualized in Fig. S7 where, d_{Au} = 10 nm nominal gold thickness, and cavity length L_{Qtz} = 82 μm).

$$S_{ref}(t) = E_{inc}(t) * h_{\text{GaP}}(t),\;\; S_{tr}(t) = E_{\text{tr}}(t) * h_{\text{GaP}}(t) = E_{inc}(t) * T_{\text{THz}}(t) * h_{\text{GaP}}(t) \qquad S5.1$$

$$\frac{E_{\text{tr}}(\omega; L_{\text{Qtz}}, d_{\text{Au}})}{E_{inc}(\omega)} = T_{\text{THz}}(\omega) = \frac{t_{\text{ext}}(\omega; d_{\text{Au}})t_{\text{int}}(\omega; d_{\text{Au}})}{1 - r_{\text{int}}^2(\omega; d_{\text{Au}})e^{-i\omega\tau_{\text{RT}}(\omega; L_{\text{Qtz}})}} \qquad S5.2$$

We apply a noise-filtering window (black curve in Fig. S7d) to suppress the sizeable noise at high THz frequencies arising from division by the reference spectra. By Fourier-transforming back to the time domain we identify the time-domain transmission function for the cavity (orange) and we can readily compare it to the intra-cavity electric field (blue) in Fig S7e. We note that the difference between the transmission coefficient and the internal, forward-propagating field is only the internal transmission coefficient, as remarked in discussion of the main text and discussed quantitatively next.

By comparison of the time-domain transmission with the intra-cavity field we can identify an approximately six-fold reduction in the transmitted field strength. The reduction is likely in fact larger, as the intra-cavity peak field strength is higher than evidenced in the chosen EOC data, where the higher frequency modes are omitted due to the large detrimental phase mismatch present at the higher frequencies in this thicker quartz EOC. The difference between the internal cavity field and the transmitted cavity field strengths is theoretically given by the internal transmission coefficient, $t_{\text{int}}(\omega; d_{\text{Au}})$, which according to our Drude modelling is $t_{\text{int}}(\Omega_{\text{THz}}; 10\text{ nm}) \cong 0.14$. This corresponds to a reduction of a factor of approximately 7, which gives good agreement with our experimental observation.

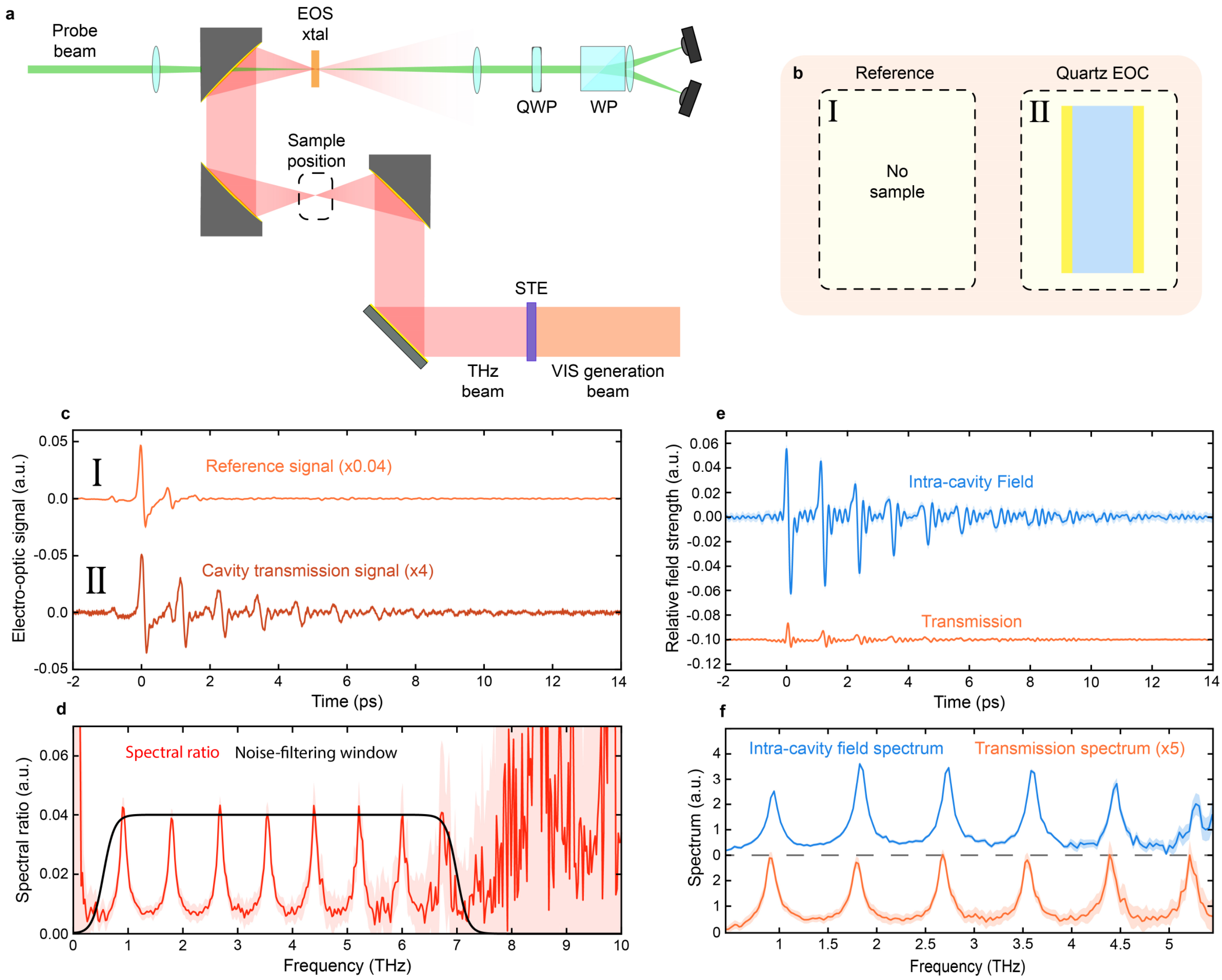

Fig. S7| THz Transmission Studies of Quartz Cavities & Comparison with EOC Sampling. a, Experimental diagram depicting the two-focus THz transmission setup. The 1st THz focus hosts the sample. The 2nd focus is used to measure the transmitted THz field using electro-optic sampling (here using 30 μm GaP; TeraSpinTec) **b**, The sample position can host no sample (reference trace) or the Quartz EOC (here d_{Au} = 10, L_{Qtz} = 82 μm). **c,** Time-domain signals for the reference and cavity transmission measurements. **d,** Division of the spectracorresponding to measurements shown in panel c yields the experimental cavity transmission function, to which we apply a noise-filtering window. **e,** Direct (numerical) comparison of the intra-cavity field measured using the cavity correction function, and the (time-domain) transmission, for the same quartz EOC. The relative strength of the intra-cavity field is depicted here by taking the peak field strength of the intra-cavity field as a ratio with the incident field peak field strength (see **Fig. 2**). For the case of the transmittance this ratio is taken automatically as a consequence of the analysis procedure. **g**, The spectra corresponding to panel **f** are shown.

Supplementary Discussion 6

Probe Scattering – Field Calibration

For the purposes of measuring the quantitative field strength, it is necessary to know the field strength of the probe laser inside of the electro-optic medium. This is typically achieved by measuring the intensity of the probe laser after the sample, and then using the refractive index of the electro-optic crystal to infer the field strength inside the crystal. In the case of an EOC, however, there is scattering and near-field optical effects for the probe pulse at the gold islands that must be considered in order to properly calibrate the probe fields which give rise to the cavity electro-optic signal.

We assume that there are losses to the probe beam intensity, whether due to scattering or reflection, which are proportional to the incident intensity, leading to the following equations for the intensity inside the EOC, $\mathrm{I_{EOC}}$, and in the transmission region $\mathrm{I_{tr}}$, for a given incident optical probe intensity $\mathrm{I_o}$ and nominal gold film thickness d_{Au} (assuming that there is no film established, i.e. purely gold islands):

$$\mathrm{I_{EOC}}(d_{\mathrm{Au}}) = t_{\mathrm{Au}}(d_{\mathrm{Au}})t_{\mathrm{ext}}(d_{\mathrm{Au}} = 0)\mathrm{I_o}, \qquad \mathrm{I_{tr}} = t_{\mathrm{Au}}^2(d_{\mathrm{Au}})\, t_{\mathrm{int}}(d_{\mathrm{Au}} = 0)\, t_{\mathrm{ext}}(d_{\mathrm{Au}} = 0)\, \mathrm{I_o} \tag{S5.1}$$

Here we have called the transmission through the gold islands t_{Au}, and the additional transmission coefficients $t_{\mathrm{ext}}(d_{\mathrm{Au}} = 0)$ and $t_{\mathrm{int}}(d_{\mathrm{Au}} = 0)$ are the terms defined in Supplementary Discussion 1. We assume that the entire optical response, prior to the establishment of gold films is attributed to scattering, or generally near-field interactions of the probe with gold islands, such that by comparing the measured probe intensities as a function of nominal gold film thickness we can infer the transmission through a single gold film:

$$t_{\mathrm{Au}}(d_{\mathrm{Au}}) = \sqrt{\frac{\mathrm{I_{tr}}(d_{\mathrm{Au}})}{\mathrm{I_{EOC}}(0)}} \tag{S5.2}$$

We compare our experimentally-inferred gold film transmittance values - using the above expression - with numerical simulations[6], where we observe very good agreement. The general trend is understood as a combination of three effects: the evolution of the scattering cross-section as the gold islands morphologies develops with nominal film thickness, the emergence of reflectivity from the partially-established gold films, and finally the evolution of the gold plasmon frequency dramatically red-shifting the spectrum of the probe pulse. The competition between these effects leads to the highly non-monotonic curve displayed in both our experimental data and the theoretical data in Fig. S7. We use these experimental transmission values to correct the field strengths that we plot in **Fig. 2e**

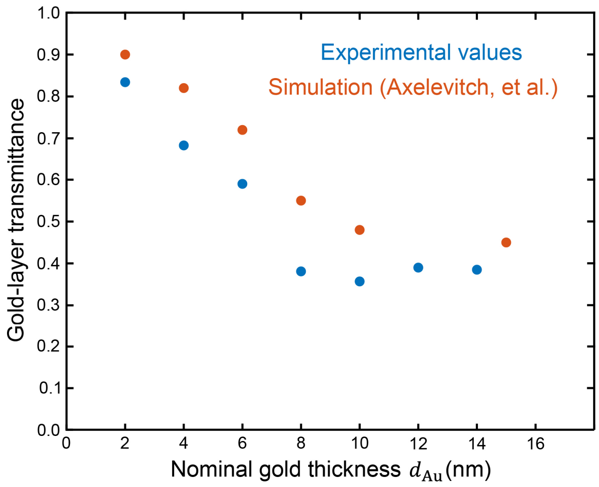

Fig. S8| Gold Island Scattering. We compare our experimentally-inferred gold island & film transmittances with simulation reported in Alexevitch et al.[6], where we have estimated the values at approximately 800 nm from their results.

Supplementary Discussion 7
Cavity Field Model

We identify the spatial functional form of the cavity electric fields by solving for the cavity eigenmodes. We consider a plane wave in each region (see Fig. S12), and impose the following boundary conditions: (1) we search for cavity modes, i.e. modes whose amplitudes go to zero at the gold interface, (2) fields which are continuous across the interfaces, and (3) fields which have a continuous derivative.

By forcing the field to go to zero at the gold boundaries (i.e. $z = \pm(L_o + L_{EO})$) we then write the fields in the left and right EO crystal as:

$$\mathrm{E}_{\mathrm{EO}}^{(\mathrm{L})}(z) = A\left(\mathrm{e}^{ik_{\mathrm{EO}}z} - \mathrm{e}^{-2\mathrm{i}k_{\mathrm{EO}}(L_o+L_{\mathrm{EO}})}\mathrm{e}^{-\mathrm{i}k_{\mathrm{EO}}z}\right) \quad (S6.1)$$

$$\mathrm{E}_{\mathrm{EO}}^{(\mathrm{R})}(z) = F\left(\mathrm{e}^{ik_{\mathrm{EO}}z} - \mathrm{e}^{2\mathrm{i}k_{\mathrm{EO}}(L_o+L_{\mathrm{EO}})}\mathrm{e}^{-\mathrm{i}k_{\mathrm{EO}}z}\right) \quad (S6.2)$$

We next enforce continuity across the air-EO crystal interfaces:

$$\mathrm{E}_{\mathrm{EO}}^{(\mathrm{L})}(z)\big|_{z=-L_o} = \mathrm{E}_{\mathrm{Air}}(z)\big|_{z=-L_o} \rightarrow 2\mathrm{i}A\mathrm{e}^{-\mathrm{i}k_{\mathrm{EO}}(L_o+L_{\mathrm{EO}})}\sin(k_{\mathrm{EO}}L_{\mathrm{EO}}) = C\mathrm{e}^{-\mathrm{i}k_o\mathrm{L_o}} + D\mathrm{e}^{\mathrm{i}k_oL_o} \quad (S6.3)$$

$$\mathrm{E}_{\mathrm{EO}}^{(\mathrm{R})}(z)\big|_{z=L_o} = \mathrm{E}_{\mathrm{Air}}(z)\big|_{z=L_o} \rightarrow -2\mathrm{i}F\mathrm{e}^{\mathrm{i}k_{\mathrm{EO}}(L_o+L_{\mathrm{EO}})}\sin(k_{\mathrm{EO}}L_{\mathrm{EO}}) = C\mathrm{e}^{\mathrm{i}k_o\mathrm{L_o}} + D\mathrm{e}^{-\mathrm{i}k_oL_o} \quad (S6.4)$$

Finally, we can enforce continuity of the first derivative:

$$\left.\frac{\partial \mathrm{E}_{\mathrm{EO}}^{(\mathrm{L})}(z)}{\partial z}\right|_{z=-\mathrm{L_o}} = \left.\frac{\partial \mathrm{E}_{\mathrm{Air}}(z)}{\partial z}\right|_{z=-\mathrm{L_o}} \rightarrow \frac{2k_{\mathrm{EO}}}{k_o}A\mathrm{e}^{-\mathrm{i}k_{\mathrm{EO}}(L_o+L_{\mathrm{EO}})}\cos(k_{\mathrm{EO}}L_{\mathrm{EO}}) = C\mathrm{e}^{-\mathrm{i}k_oL_o} - D\mathrm{e}^{\mathrm{i}k_oL_o} \quad (S6.5)$$

$$\left.\frac{\partial \mathrm{E}_{\mathrm{EO}}^{(\mathrm{R})}(z)}{\partial z}\right|_{z=\mathrm{L_o}} = \left.\frac{\partial \mathrm{E}_{\mathrm{Air}}(z)}{\partial z}\right|_{z=\mathrm{L_o}} \rightarrow \frac{2k_{\mathrm{EO}}}{k_o}F\mathrm{e}^{\mathrm{i}k_{\mathrm{EO}}(L_o+L_{\mathrm{EO}})}\cos(k_{\mathrm{EO}}L_{\mathrm{EO}}) = C\mathrm{e}^{\mathrm{i}k_oL_o} + D\mathrm{e}^{-\mathrm{i}k_oL_o} \quad (S6.6)$$

With these last four equations, we next identify solutions which satisfy all boundary conditions. By repeated substitution, we identify the following two expressions, which are the transcendental equations that can produce the possible eigenvalues.

$$\frac{k_{\mathrm{EO}}}{k_o}\tan(k_oL_o) = -\tan(k_{\mathrm{EO}}L_{\mathrm{EO}}), \quad \frac{k_{\mathrm{EO}}}{k_o}\cot(k_oL_o) = \tan(k_{\mathrm{EO}}L_{\mathrm{EO}}) \quad (S6.7)$$

Here the first equation holds only when $C - D \neq 0$, and similarly the second only when $\mathrm{C} + \mathrm{D} \neq 0$. We identify these equations then as physically referring to odd ($C = -D$) and even ($C = D$) modes, respectively. By using either of these restrictions on the relation between forward- and backwards-propagating plane waves in air, we obtain the electric field defined in Methods Eqtns. $\mathrm{M.11}$ and $\mathrm{M.12}$ from equations $\mathrm{S6.3}$ and $\mathrm{S6.4}$ above.

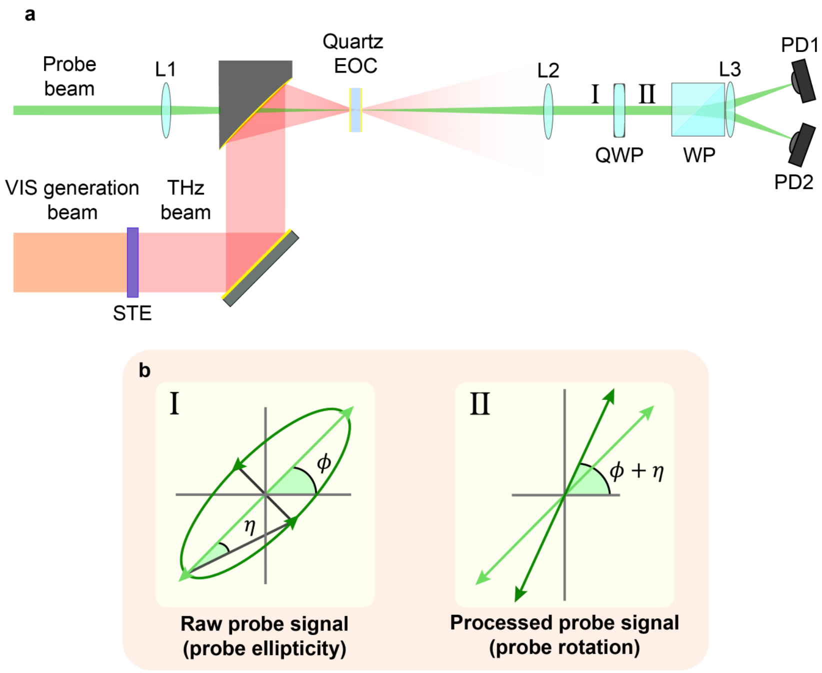

Fig. S9| Balanced Detection of Electro-Optic Cavity Signal. a, The high-power visible pulse pumps the spintronic emitter, generating a THz pulse, which is subsequently focused into the quartz EOC. The THz pulse reflects periodically inside the cavity, alongside the probe pulse co-focused into the cavity, thereby generating EOC signal via frequency-mixing process. This signal results in an effective ellipticity imparted to the probe beam pulse, which is measured using the balanced detection optics. **b**, The polarization of the probe beam after interaction in the cavity is plotted at the various stages in the balanced detection scheme. The gray lines denote the projection of the polarization state onto the x and y axes, and the lighter green vector denotes the polarization state of the probe in the absence of any THz field interaction. After THz field interaction in the EOC, the probe acquires an ellipticity, η. This ellipticity is converted into a rotation of the linear polarization angle by use of a QWP. This rotation is measured by splitting the polarization into vertical and horizontal components using the WP and measuring the difference signal from the intensities on a photodiode pair.

Figure Legend: L1, L2, L3 – Lenses 1-3, STE – Spintronic Emitter EOC – Electro-optic Cavity, QWP – Quarter Waveplate, WP – Wollaston Prism, PD1, PD2- Photodiodes 1,2

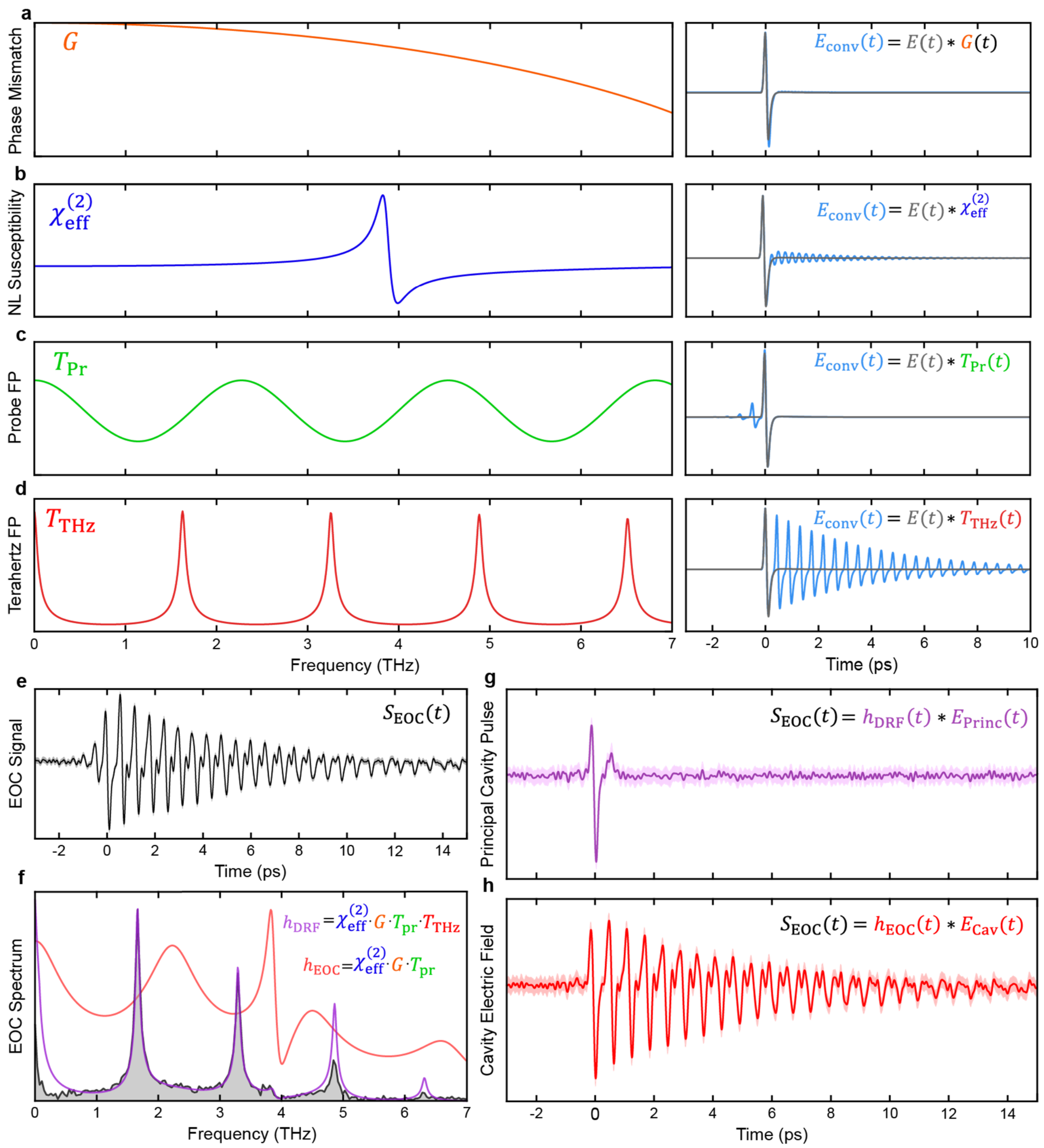

Fig. S10| Cavity Correction Function. The most important frequency-dependent components of the cavity correction function are shown as a function of terahertz frequency, for the EOC conditions investigated in **Fig. 1** For panels a-d, the left panel shows the absolute value of the function in the frequency domain, and the right side the result of convolution with a theoretical principal pulse. **a,** The absolute value of the frequency-dependent phase-mismatch (orange). **b,** The absolute value of the frequency-dependent effective 2nd order nonlinear susceptibility (blue). **c,** The absolute value of the frequency-dependent effective 2nd order nonlinear susceptibility (blue). **d,** The absolute value of the frequency-dependent cavity fields transfer function at probe frequencies (green). **e,** The absolute value of the frequency-dependent cavity fields transfer functio for THz frequencies (red). For panels a-d, plotted additionally on the right are the convolution of these (complex) functions with a model THz pulse. The nonlinear susceptibility displays the signature of a phonon resonance, the phase-mismatch displays a slight broadening of the THz pulse (more severe for thicker EOS crystals), the cavity field for the probe shows a negative-time pulse train (very low Q), and the cavity field for the THz field shows a positive-time pulse train (higher Q). **e**, Raw Experimental EOC signal, as displayed in **Fig 1**. **f**, Raw Experimental EOC spectrum (black), EOC correction function (red), and cavity detector response function (purple). The detector response function differs from the correction function by the additional term for de-convolving the Fabry-Pérot resonances for the intra-cavity THz pulse. **g**, The result of deconvolving the EOC signal with the cavity detector response function yields the principal single-cycle pulse, demonstrating appropriate choice of THz internal cavity reflectivity. **h**, Cavity electric field, resulting from deconvolving the EOC signal with the cavity correction function, as plotted in **Fig. 1**.

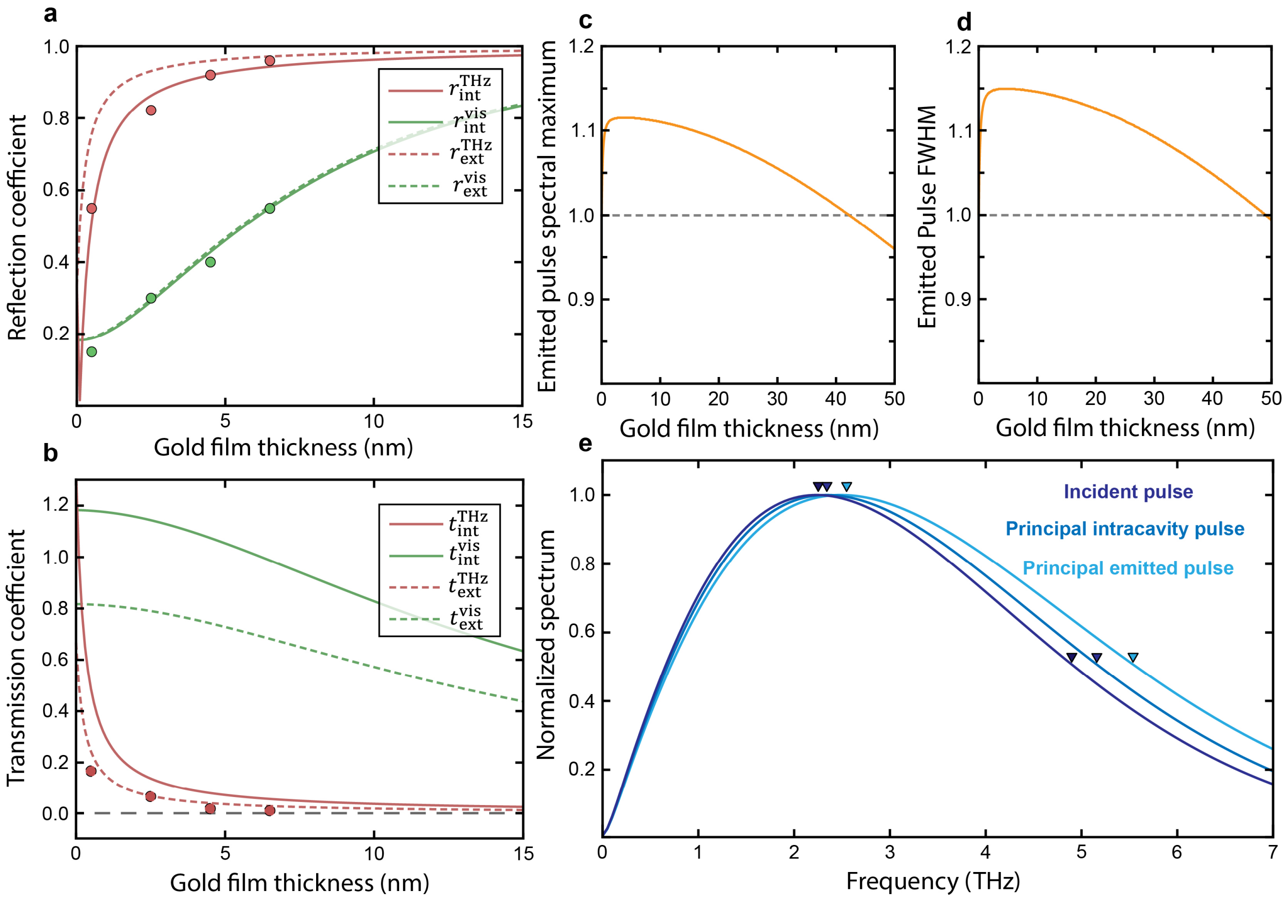

Fig. S11| Gold Film Mirror Optical Properties, & Dispersion. a,b, Field transmission and reflection coefficients for THz and visible frequencies (0.35 THz and 380 THz, here, respectively) calculated for both internal and external pulses, as defined in Supplementary information. Dots represent experimentally-inferred data, along with the results shown in **Fig. 3e**. The reflection values are identified via inspection of deconvolved cavity fields, and the transmission values from the resulting cavity peaks fields, after treatment using the numerical cavity correction function. All experimental reflectivity values are identified independently of the thin-film electromagnetic model. **c,d,** Using a theoretical THz pulse and the electromagnetic three-medium model, we compute emitted pulses for various different gold film thicknesses. In panel c we show the frequency of the emitted pulse's strongest spectral weight. In panel d, we show extracted FWHM of the emitted pulse. **e,** The normalized spectrum of the incident pulse, principal intra-cavity pulse, and principal emitted pulse, for d_{Au} = 5 nm. The spectral maximum and FWHM frequency are denoted for each pulse with a colored triangle.

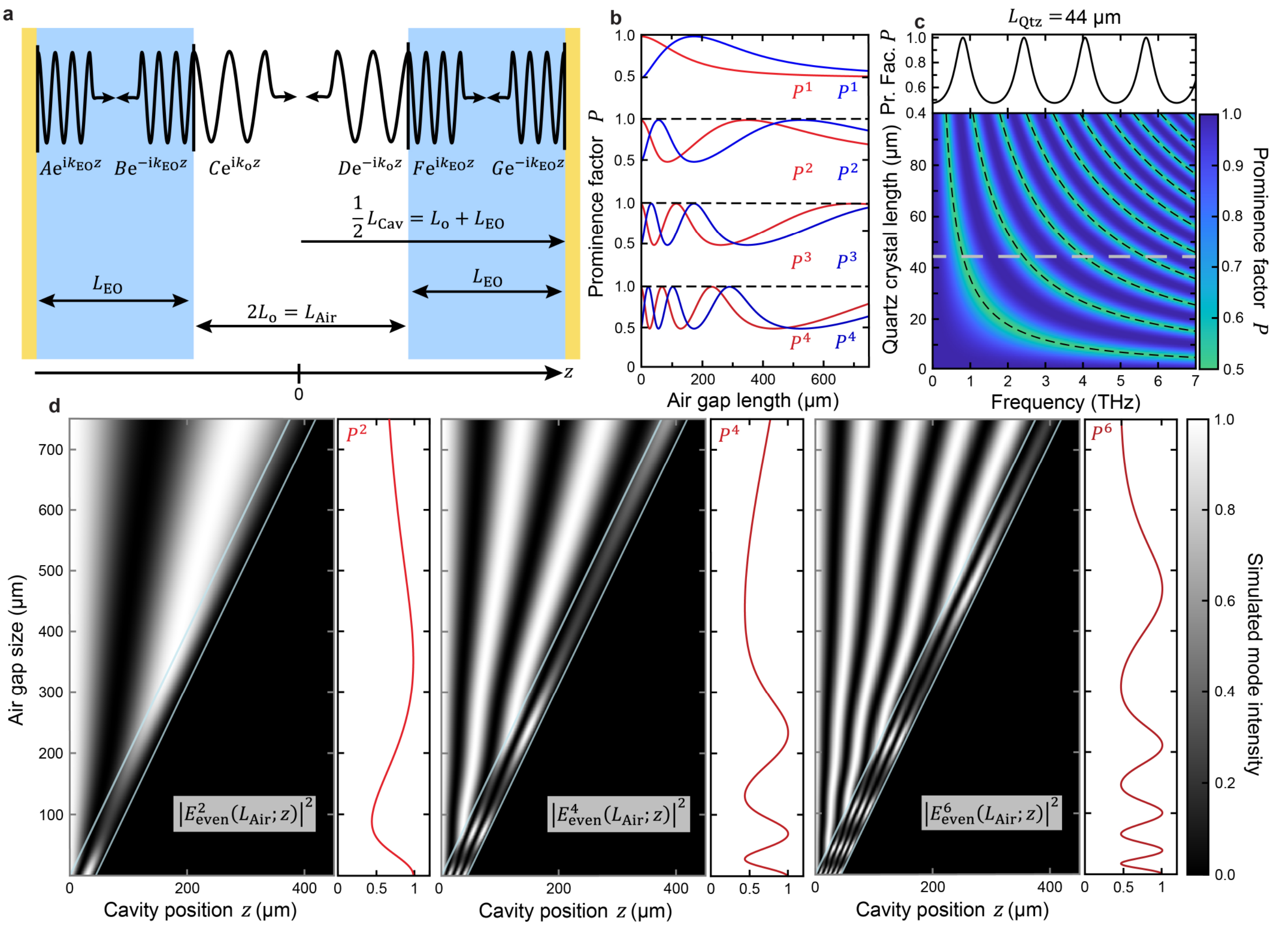

Fig. S12| Cavity-Field Model. a, Formulation of the hybrid EOC cavity-field model. In each section there is a forward- and backwards-propagating planewave. The wavevector in the electro-optic crystal and air is dictated by the refractive index at the corresponding frequency in either layer. **b,** The prominence factor, depicted for the first 4 even (red) and odd (blue) modes, as a function of the air gap length, simulated for $n_{EO} = 2$. **c,** The prominence factor is plotted as a function of the quartz crystal length (lower panel), and for the specific quartz thickness experimentally implemented here L_{Qtz} = 44 μm (upper panel). Both panels depict the prominence factor in the absence of dispersion, using $n_{EO} = n_{Qtz} = 2.1$. **d,** Intensity profiles of the 2nd, 4th, and 6th even cavity modes, as a function of the air gap length. Only positive spatial positions are displayed, as the intensity for all modes has even mirror symmetry. The boundaries of the quartz are depicted in all plots with the light blue lines. For each intensity plot, the associated integrated intensity ratio and prominence factors are displayed to the right side of the false-color plot, which demonstrate local maxima wherever the amplitude in quartz is maximized.

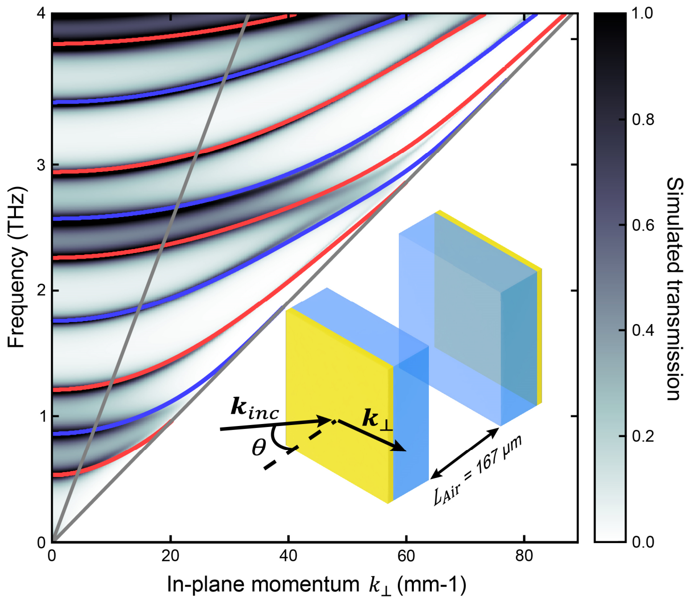

Fig. S13| Cavity Momentum Dispersion. Depiction of the non-normal incidence generalized cavity field model eigenvalues, colored red (even) and blue (odd) in reference to the parity symmetry. These eigenvalues are overlaid onto SMM-simulated transmittance. Both simulations are computed for L_{Qtz} = 44 μm and L_{Air} = 167 μm. We depict the light line corresponding to grazing incidences (exterior light line), and corresponding to an incidence angle of 22.5° (interior light line). Based on the beam size and focal length of the parabolic mirror, the majority of our THz radiation lies within this interior light line. We observe that there is minimal dispersion of the modes within this region, thus indicating that momentum dispersion doesn't play a large role in our hybrid EOC measurements.

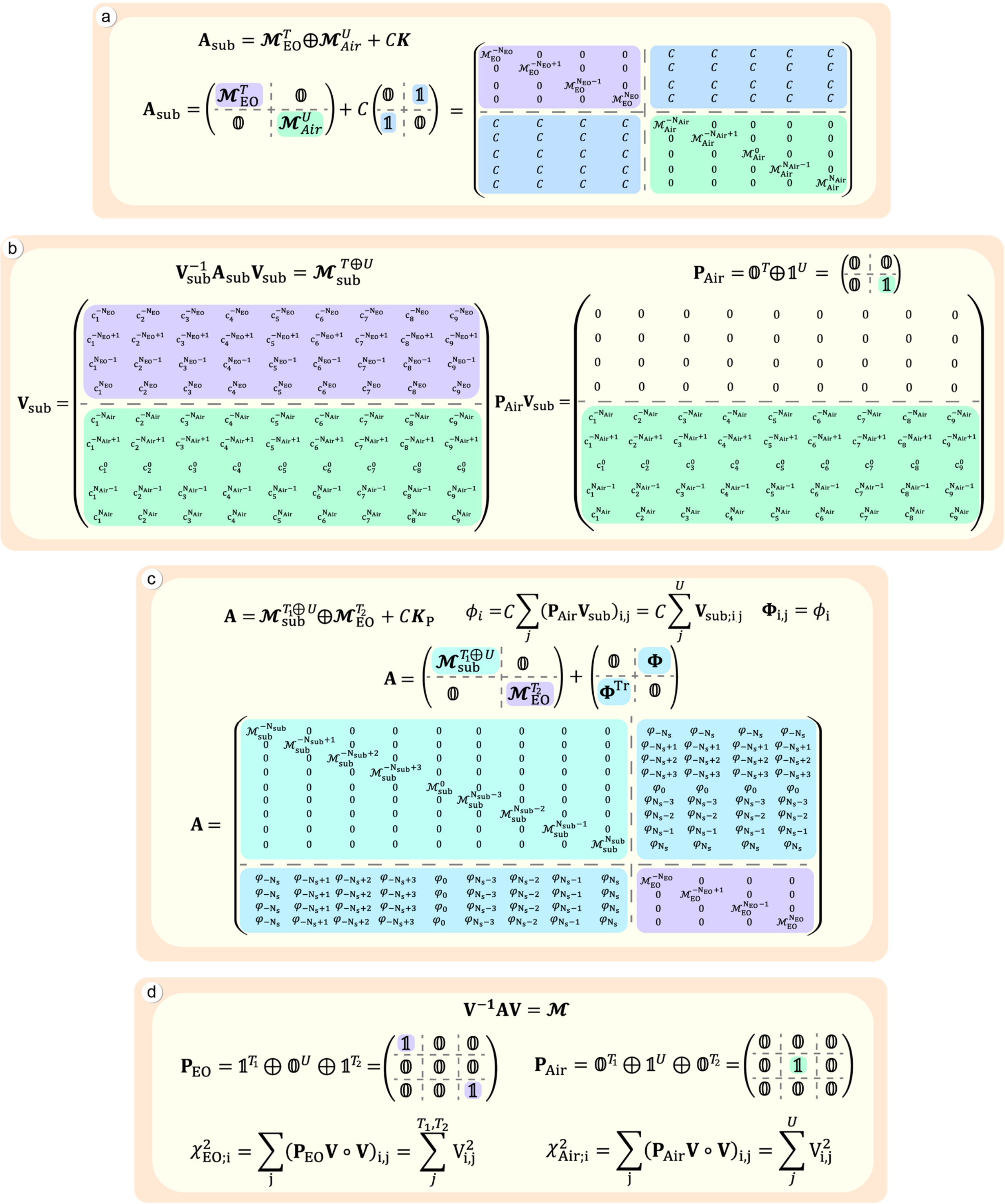

Fig. S14| Coupled-Oscillator Model – Coupling Matrix Formalism. a, Depiction of the coupling matrix for the sub-cavity, coupling one EO crystal with the air gap. It is represented here using $\mathrm{N_{Air}} = \mathrm{N_{EOC}}$ = 2, resulting in a matrix dimension of $\mathrm{N_{sub}} = (2\mathrm{N_{Air}} + 1) + 2\mathrm{N_{EO}}$ = 9, partitioned into the blocks filled by the original resonances, and the coupling sub-blocks. **b,** The eigenvectors from the sub-cavity coupling matrix are depicted, as well as the result from projection onto the air sub-space, as is needed to construct the coupling matrix for the full cavity, as shown in panel c **c,** The full coupling matrix is depicted, which is built from the sub-blocks of the final EO crystal and the resultant eigenvalues from the sub-cavity, where these two blocks are then coupled via the same coupling matrix as in the sub-cavity, excepted for a modification by the integrated projection of the sub-cavity eigenvectors onto the air sub-space. We term the matrix collecting these integrated projections times the coupling constant $\boldsymbol{\Phi}$, where for the ith eigenvector of the sub-cavity system, its integrated projection onto the air sub-space is then ϕ_i/C. **d,** The character of the final resulting eigenvectors is calculated either by using the projection operator onto any particular sub-space on the matrix consisting the square of the eigenvectors (i.e. the Hadamard product of $\mathbf{V}$ with itself), or equivalently by performing the summation over the squared elements of the matrix $\mathbf{V}$ within the appropriate sub-space.

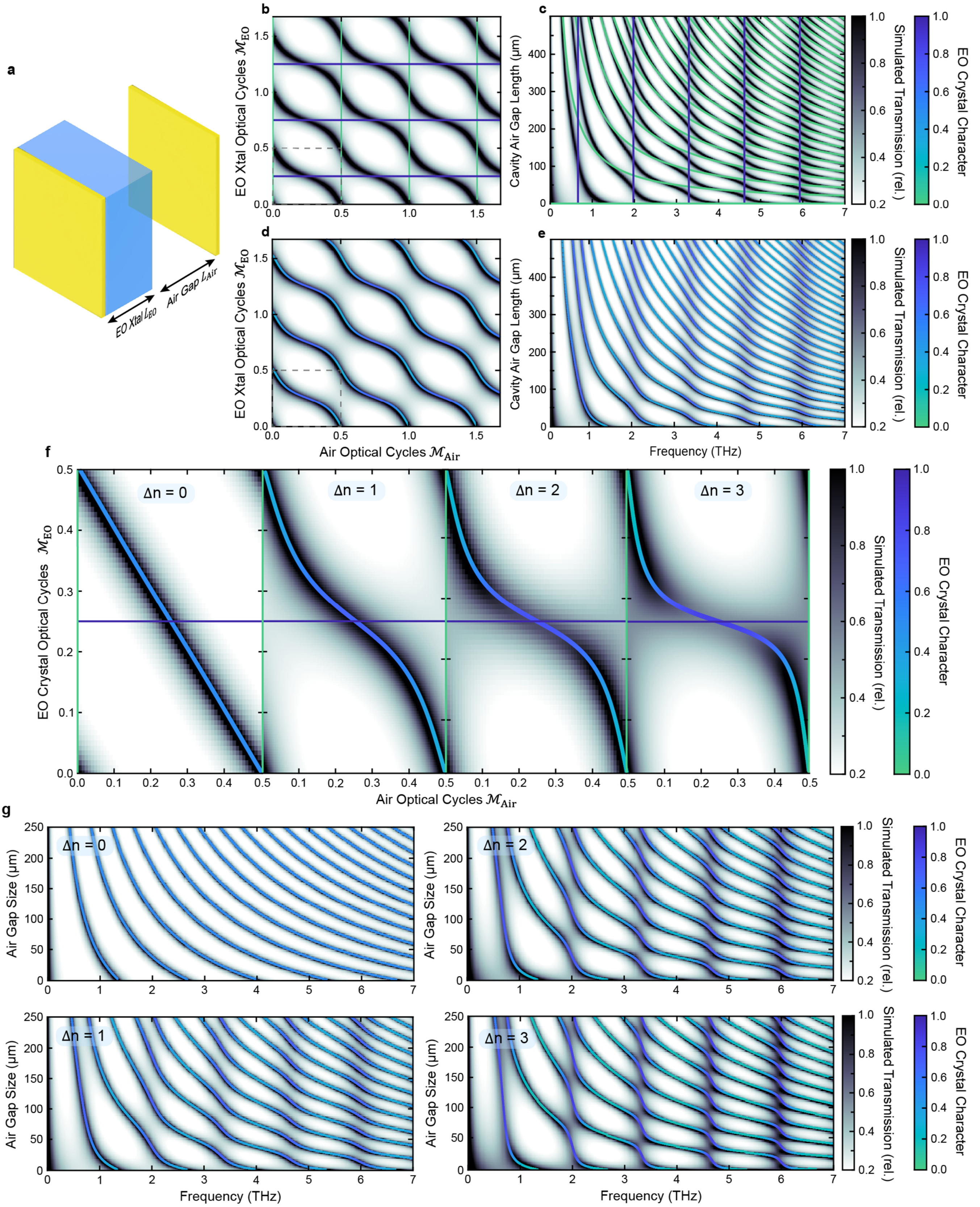

Fig. S15| Coupled-Oscillator Model – Partial Cavity. **a,** Depiction of the partial cavity analyzed here, where there is a single EO crystal of thickness L_{EO} and two gold mirrors, separated by a variable-size air gap L_{Air}. **b,** Simulated transmission as a function of number of optical cycles in air and the EO crystal, for n_{EO} = 2, along with the basis resonances, colored according to the EO character. The irreducible space is denoted by the dashed gray square. **c,** Simulated transmission as a function of number of air gap size and THz frequency, for n_{EO} = 2, along with the corresponding basis resonances, colored according to the EO character. **d,** The eigenvalues are plotted on top of the simulated transmission displayed in panel b, colored here according to the resultant EO character. **e,** The eigenvalues are plotted on top of the simulated transmission displayed in panel c, colored according to the resultant EO character. **f,** The eigenvalues are plotted in the irreducible space, as a function of EO crystal refractive index, referenced here as a difference according to the air refractive index of unity, highlighting maximal coupling at no index mismatch, and a shrinking coupling magnitude as the refractive index mismatch becomes larger. **g,** A representative cavity dispersion is plotted for each sub-panel in panel f, where the optical thickness has been normalized, to highlight the behavior of the mode coupling strength as a function of varying refractive index. In each sub-panel, the simulated cavity transmission is displayed in gray, and the eigenvalues obtained from the coupled-oscillator model are displayed, and colored according to the EO crystal character.

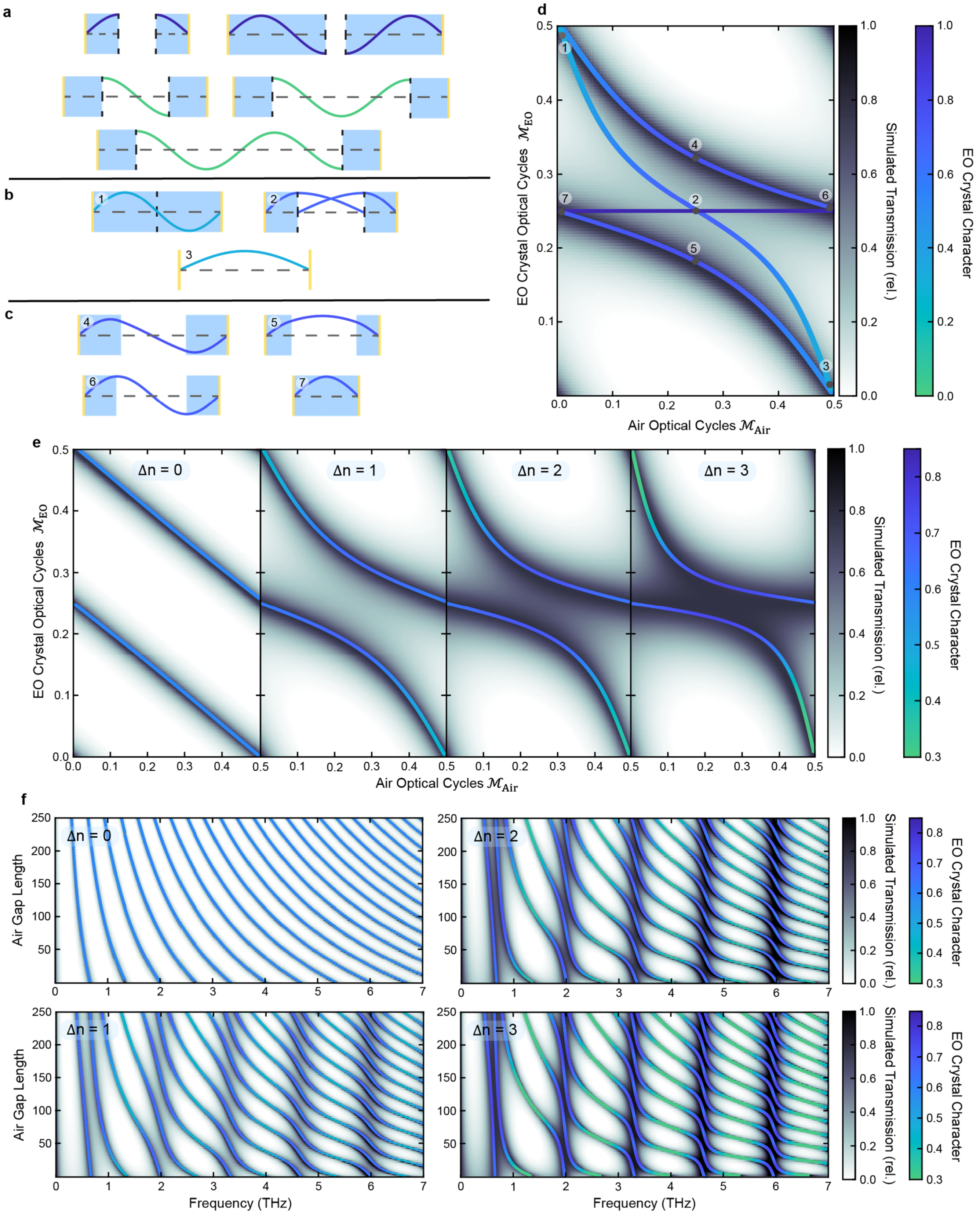

Fig. S16| Coupled-Oscillator Model – Full Cavity. a, $\lambda/4$ standing-wave resonances in EO crystals (purple) and $\lambda/2$ standing-wave resonances in the air gap (green), all for a common frequency, with variable EO crystal lengths (top) and air gap sizes (bottom). **b,** Selected resonance conditions derived from the sub-cavity eigenvectors, colored according to the EO character (scale in panel d), and depicted at illustrative cavity conditions, corresponding to labels in panel d. **c,** Resonance conditions corresponding to the full-cavity eigenvectors, colored according to the derived EO character (scale in panel d), depicted at illustrative cavity conditions labelled in panel d. **d,** Simulated field transmission (false color, gray), along with resonances corresponding to the sub-cavity (interior eigenfunction), and the symmetric (below) and anti-symmetric (above) eigenvectors, colored according to the corresponding quartz character. **e,** The simulated transmission for a series of EO crystal refractive indices, referenced according to refractive index of air (n_{Air} = 1), to demonstrate the relative 'de-coupling' of the air and EO-crystal modes for high refractive indices. **f,** The frequency dispersion of the optical modes is shown for the refractive indices corresponding to panel e, representing the evolution of the coupling strength as it would be observed experimentally.

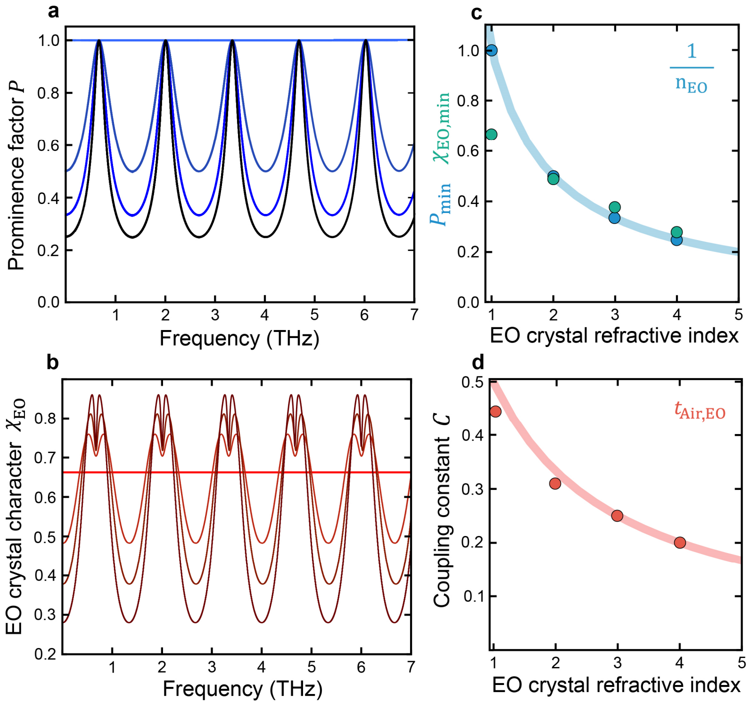

Fig. S17| Effect of Electro-Optic Crystal Refractive Index. a, The prominence factor is depicted, for the electro-optic crystal refractive indices of 1,2,3,4 with decreasing saturation. **b,** The electro-optic crystal character identified from the coupled-oscillator model are plotted for the same input refractive indices as considered in panel a. **c,** The coupling constant identified from the coupled-oscillator model is plotted for the various EO crystal refractive indices that were investigated, and compared with the transmission coefficient from the air gap into the EO crystal, displaying very good correspondence. **d,** The minimum value observed in both the prominence factor and EO crystal character are displayed as a function of EO crystal refractive index, where we note that the computed prominence factor minimum is exactly equal to the inverse of the refractive index, whereas the EO crystal character follows a very similar trend.

Table S1| Quartz Dielectric Function

Values adapted and expanded from Frenzel, et.al.

$$\varepsilon(f) = 2.103 + \sum_l \frac{A_l f_l^2}{f_l^2 - f^2 - i\Gamma_l f}$$

Mode Index	Central Frequency f_l (THz)	Amplitude A_l	Damping Factor Γ_l (THz)
**Oscillator 1**	3.85	0.000055	0.09
**Oscillator 2**	7.97	0.032	0.167
**Oscillator 3**	11.76	0.37	0.096
**Oscillator 4**	13.45	0.75	0.177
**Oscillator 5**	24	0.14	0.6
**Oscillator 6**	37.2	0.6	0.6

Table S2| Quartz 2nd Order Nonlinear Susceptibility

Values adapted from Frenzel, et.al., where available, and otherwise inferred via standard electro-optic measurements.

$$\chi^{(2)}_{eff}(f) = 0.28 \cdot \left(1 + \sum_l \frac{C_l f_l^2}{f_l^2 - f^2 - i\Gamma_l f}\right) \text{ [pm V-1]}$$

Mode Index	Central Frequency f_l (THz)	Faust-Henry Coef. C_l	Damping Factor Γ_l
**Oscillator 1**	3.85	0.11*	0.09
**Oscillator 2**	7.97	-0.012	0.167
**Oscillator 3**	11.76	0.5	0.096
**Oscillator 4**	13.45	-0.6	0.177
**Oscillator 5**	24	1.2	0.6

* C_1 found to vary widely from crystal to crystal, by up to nearly 50%

References

1. Ismail, N., Kores, C. C., Geskus, D. & Pollnau, M. Fabry-Pérot resonator: spectral line shapes, generic and related Airy distributions, linewidths, finesses, and performance at low or frequency-dependent reflectivity. *Optics Express* 24, 16366 (2016).
2. Frenzel, M. *et al.* Quartz as an Accurate High-Field Low-Cost THz Helicity Detector. *Optica* **11**, 362 (2024).
3. Kampfrath, T., Nötzold, J. & Wolf, M. Sampling of broadband terahertz pulses with thick electro-optic crystals. *Applied Physics Letters* 90, 231113 (2007).
4. Shen, Y.R. *The Principles of Nonlinear Optics*. (New York: Wiley, 1984).
5. Gallot, G. & Grischkowsky, D. Electro-optic detection of terahertz radiation. *Journal of the Optical Society of America B* **16**, 1204-1212 (1999).
6. Axelevitch, A., Apter, B. & Golan, G. Simulation and experimental investigation of optical transparency in gold island films. *Optics Express* **21**, 4126 (2013).
